# Supplementary figures and images for: Chondroitin sulfate proteoglycan 4,6 sulfation regulates sympathetic nerve regeneration after myocardial infarction
Source: eLife. 2022 May 23;11:e78387. doi: 10.7554/eLife.78387 (PMC9197393; doi:10.7554/eLife.78387)

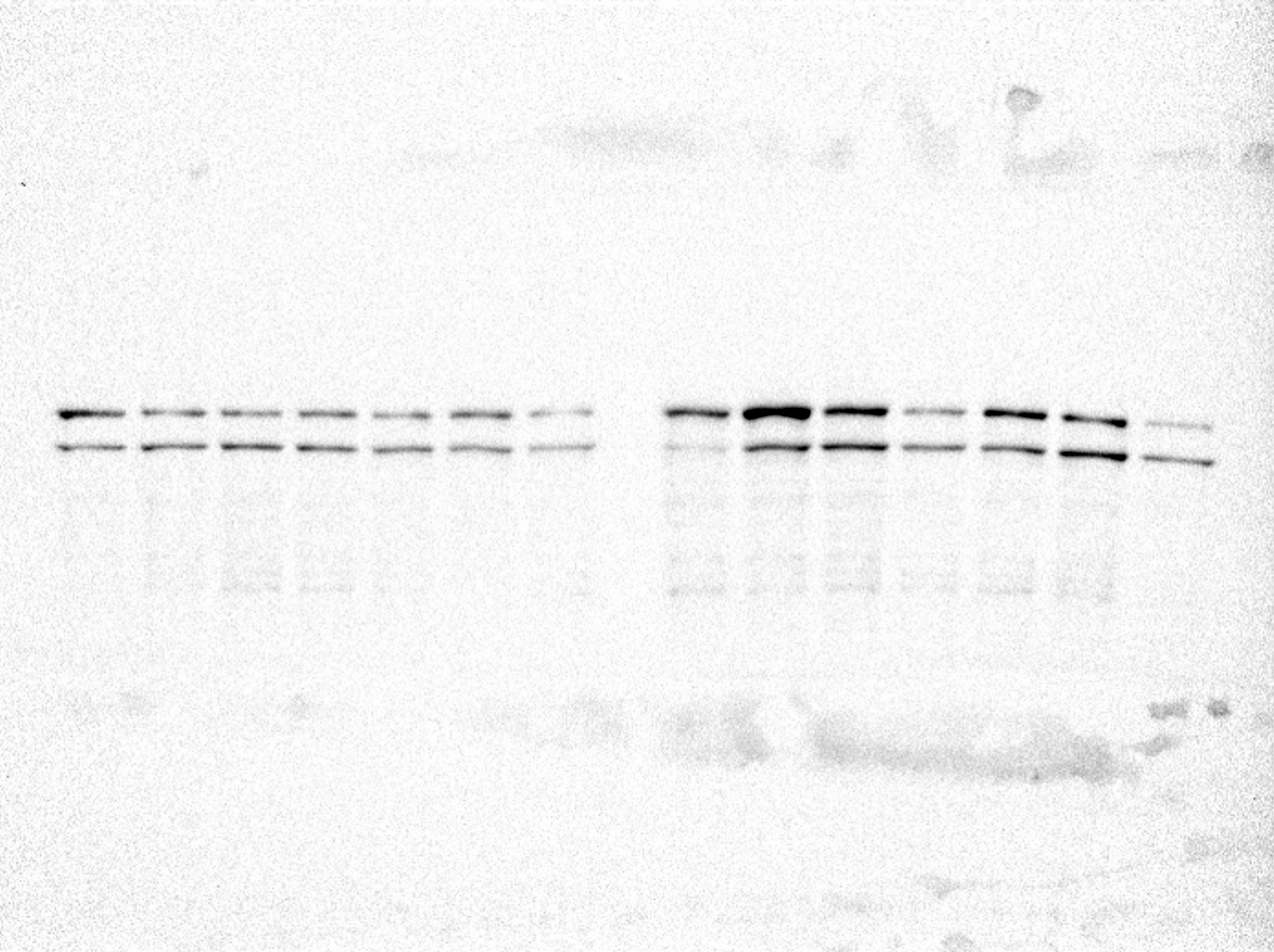

Supplement: Source data 1. [file elife-78387-data1.zip › Western blot source data/raw files/Figure 6 - TH.tif]

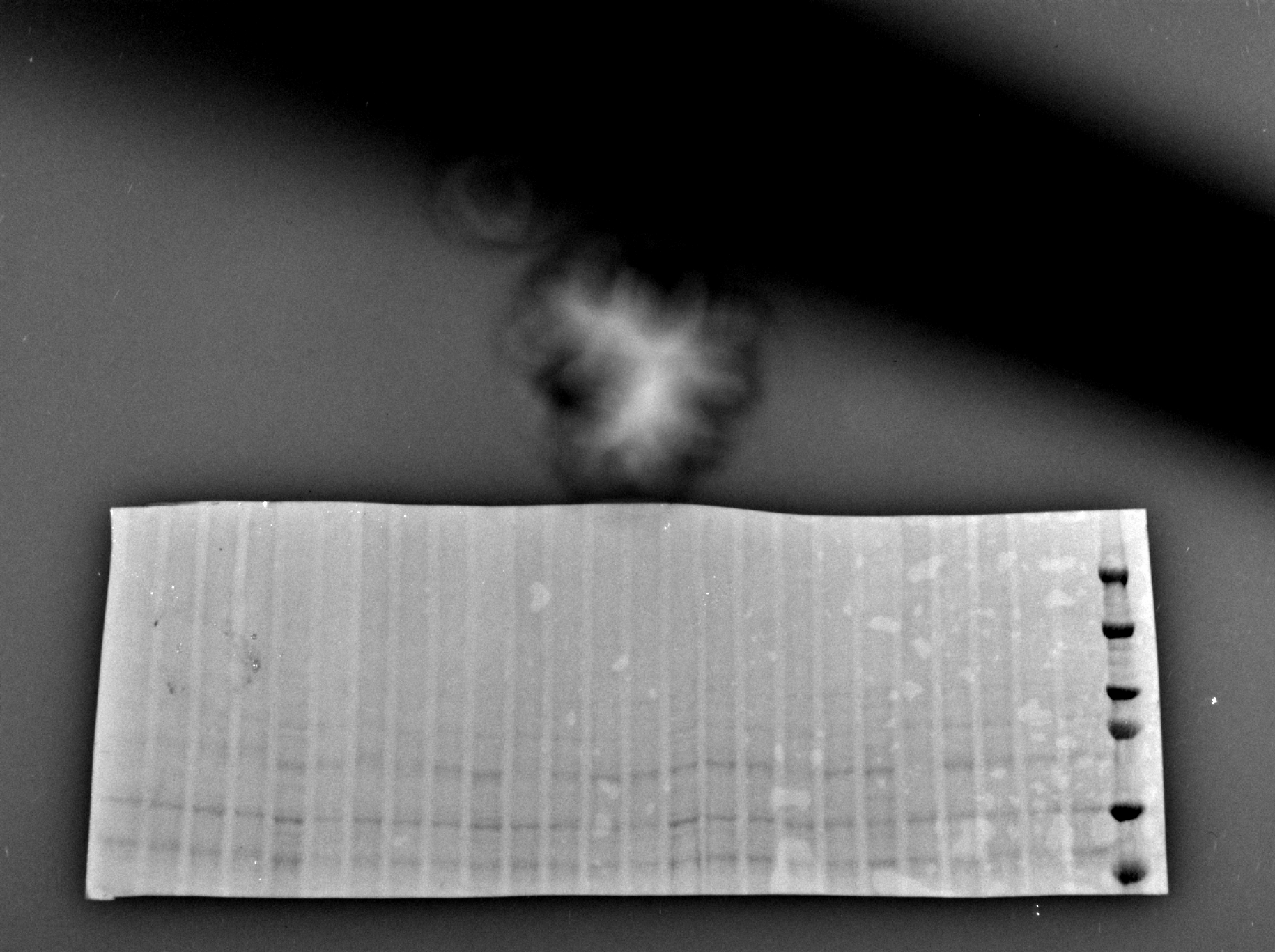

Supplement: Source data 1. [file elife-78387-data1.zip › Western blot source data/raw files/Figure 5 - ponceau.tif]

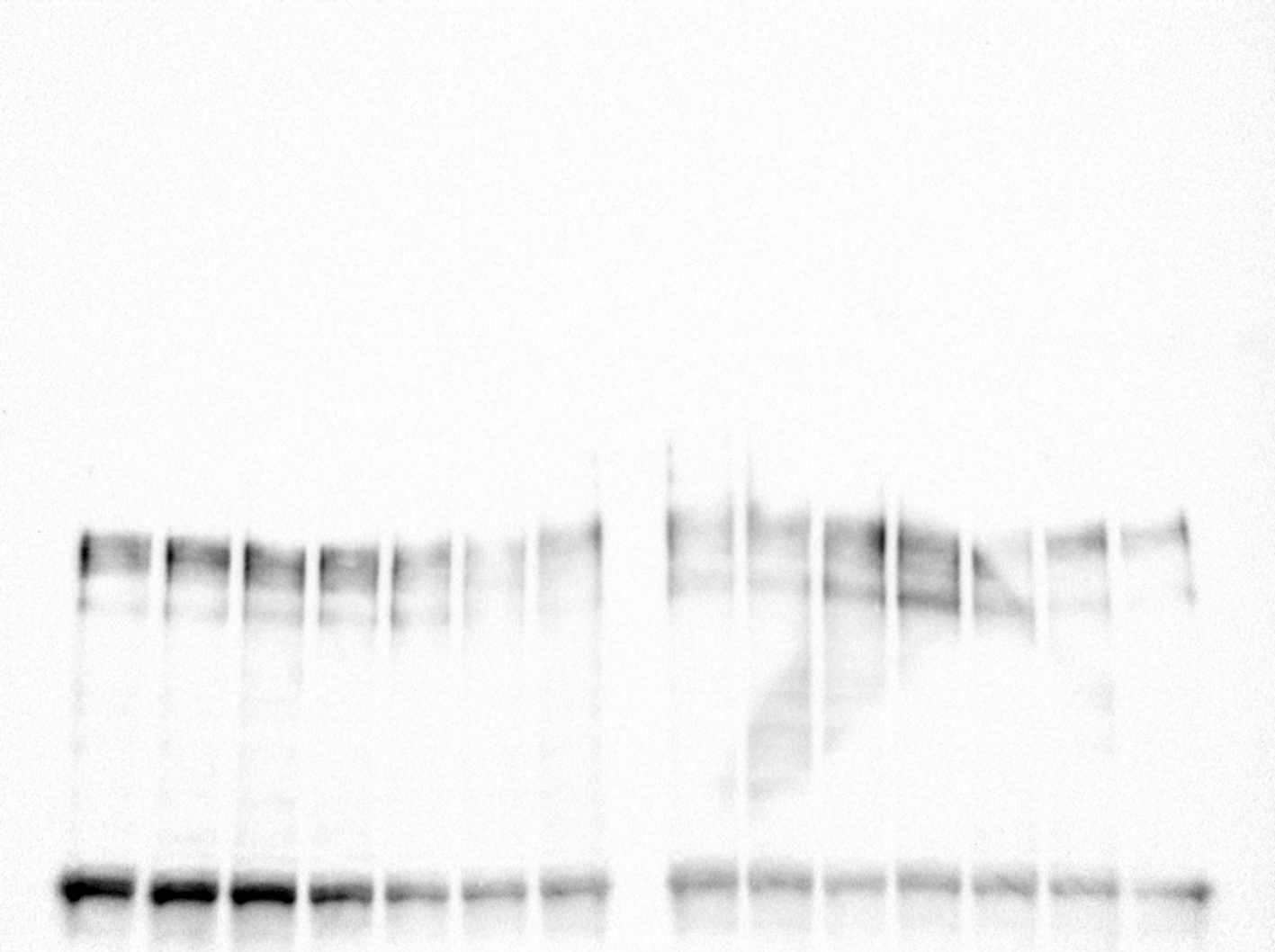

Supplement: Source data 1. [file elife-78387-data1.zip › Western blot source data/raw files/Figure 6 - 4S-CS GAGs.tif]

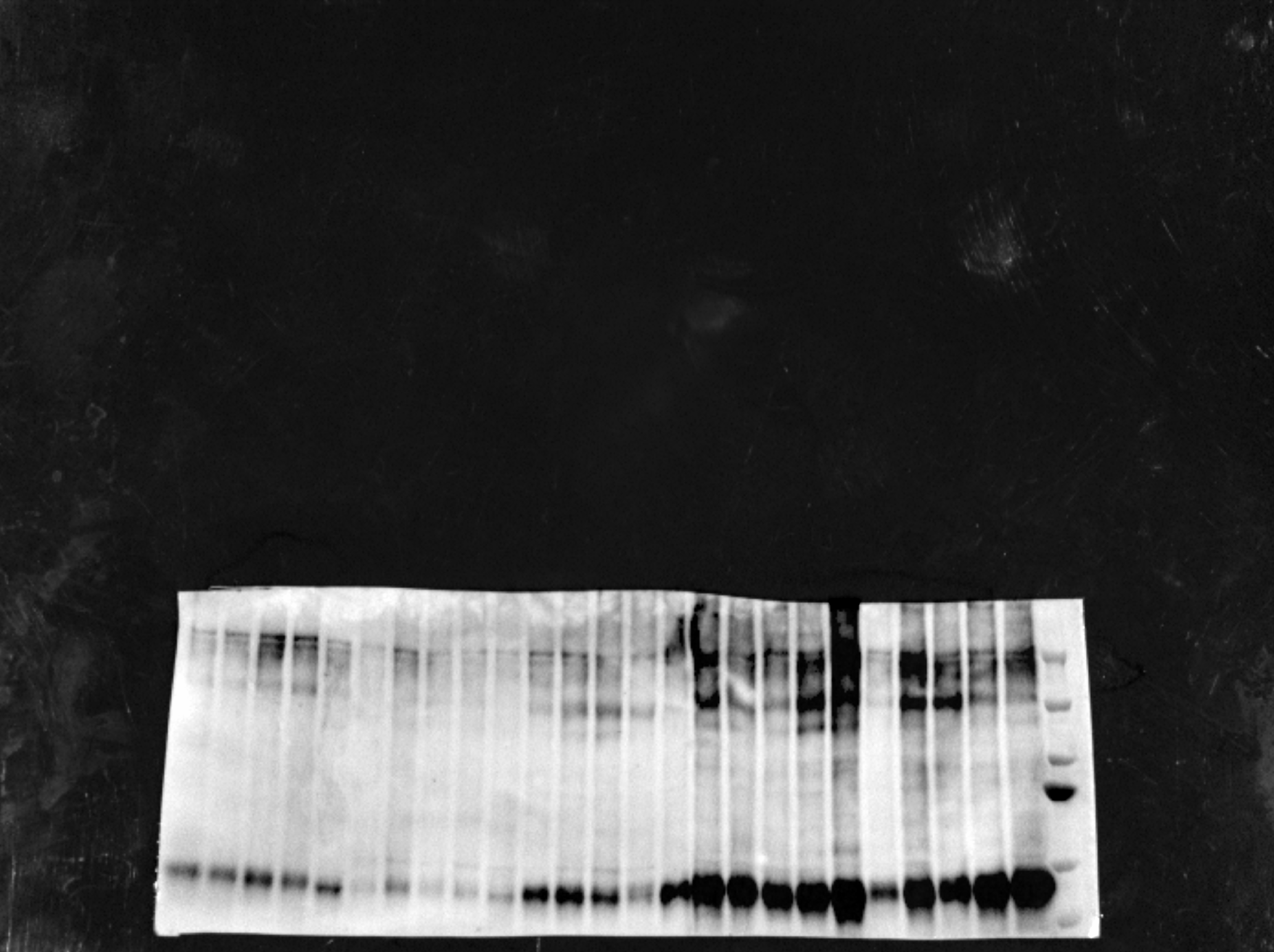

Supplement: Source data 1. [file elife-78387-data1.zip › Western blot source data/raw files/Figure 5 - 6S-CS GAGs.tif]

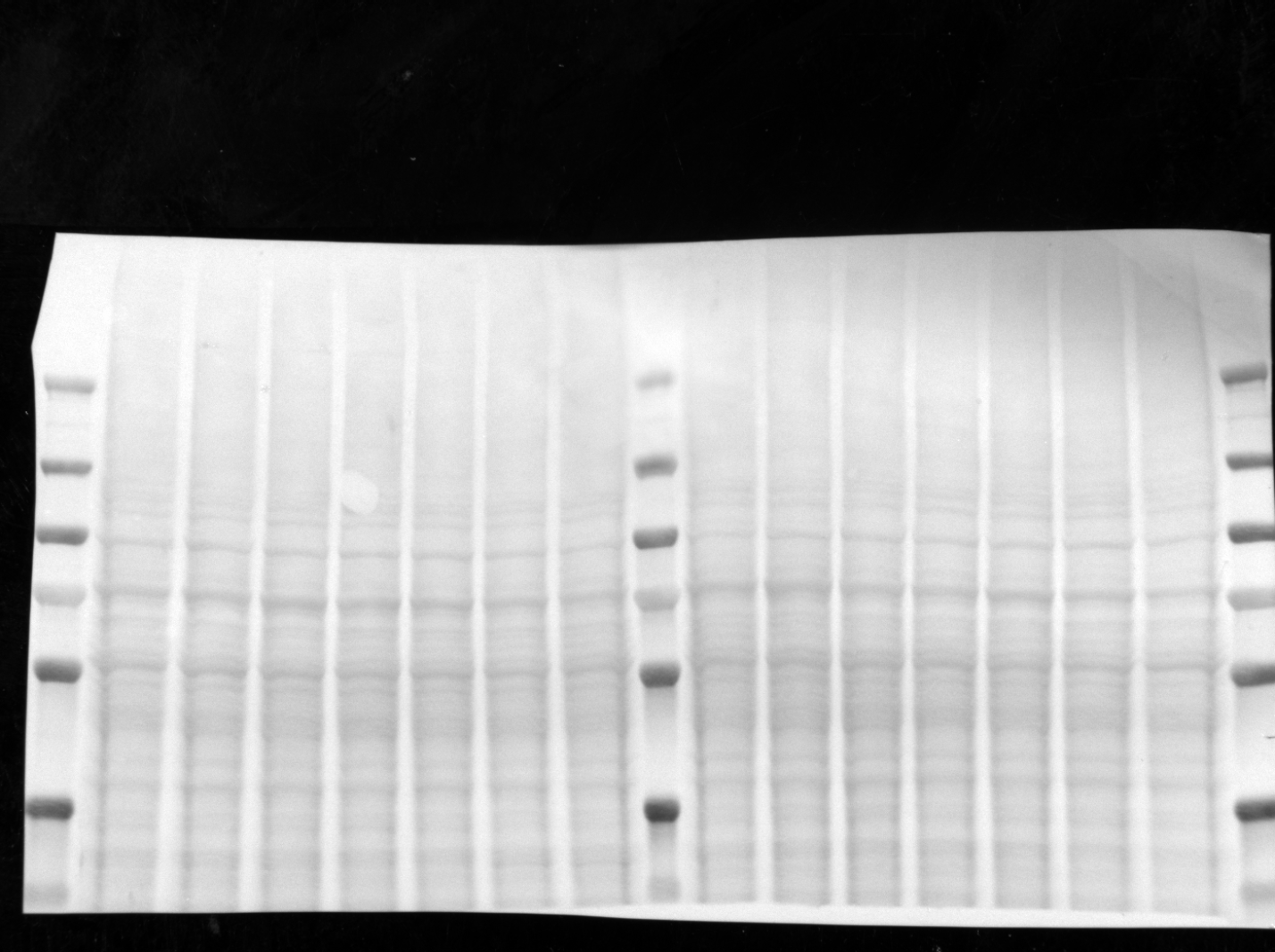

Supplement: Source data 1. [file elife-78387-data1.zip › Western blot source data/raw files/Figure 6 - ponceau.tif]

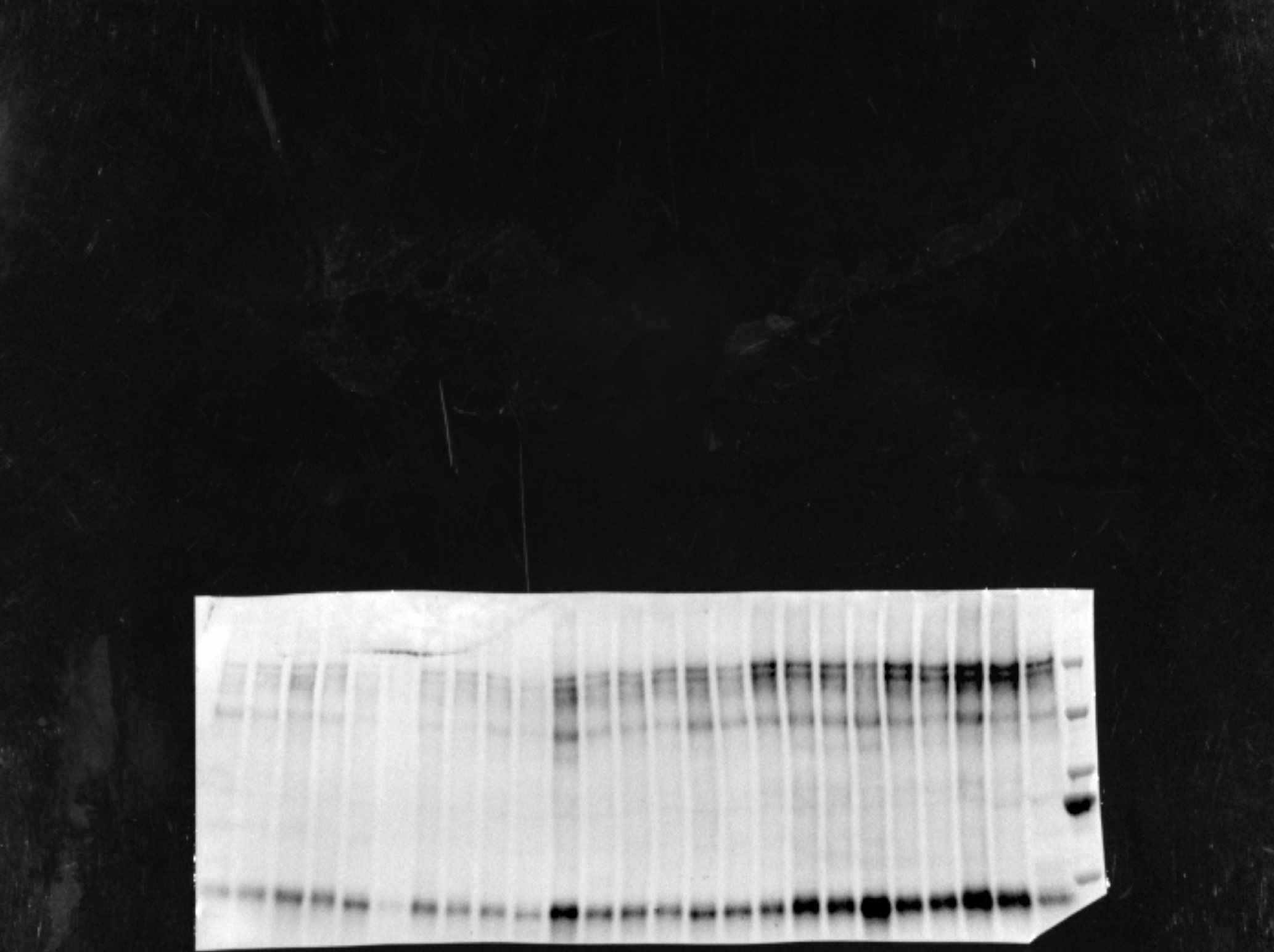

Supplement: Source data 1. [file elife-78387-data1.zip › Western blot source data/raw files/Figure S1 - non-scar 6-sulfation.tiff]

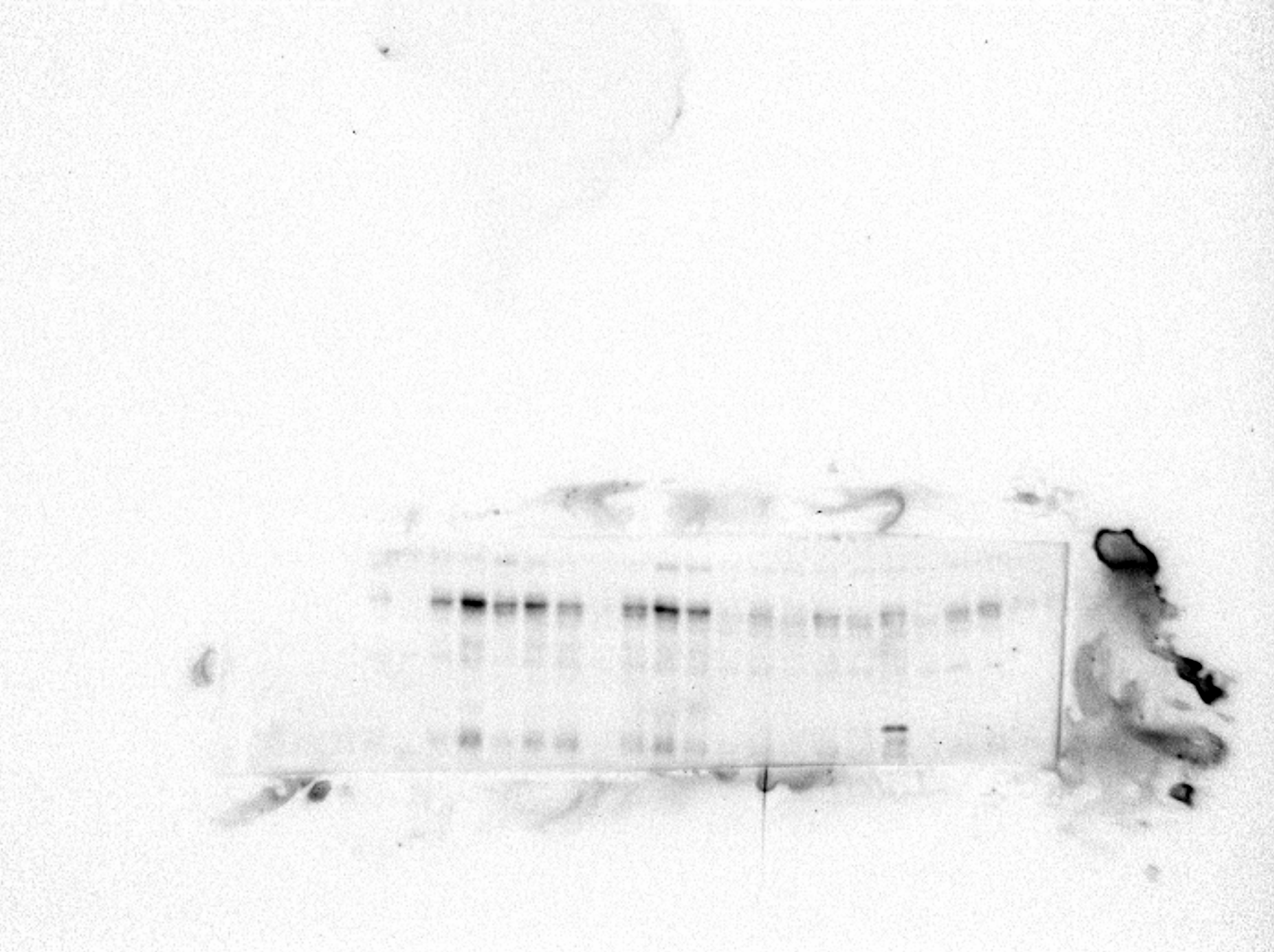

Supplement: Source data 1. [file elife-78387-data1.zip › Western blot source data/raw files/Figure 5 - Galectin-3.tif]

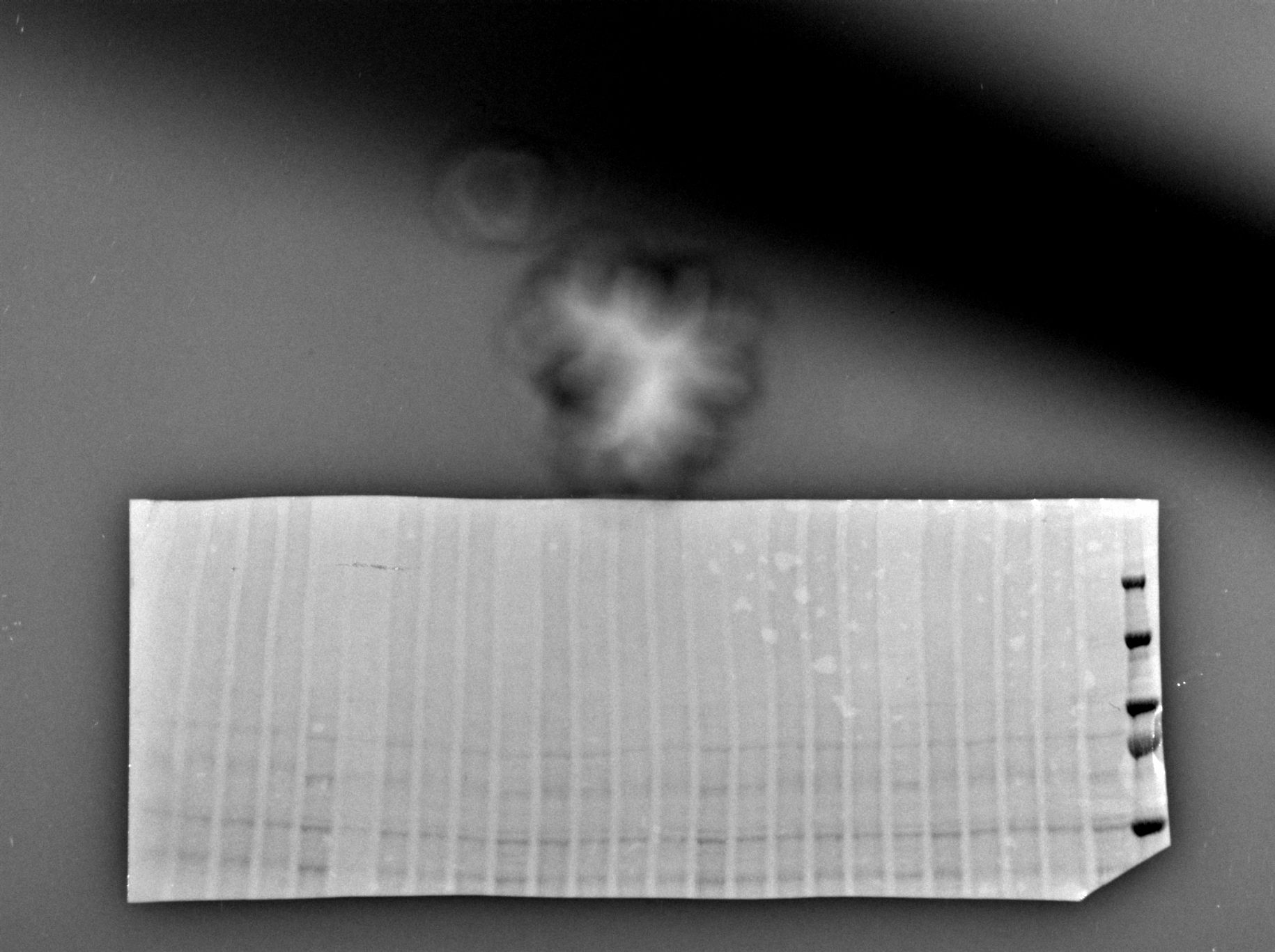

Supplement: Source data 1. [file elife-78387-data1.zip › Western blot source data/raw files/Figure S1 - non-scar ponceau.tiff]

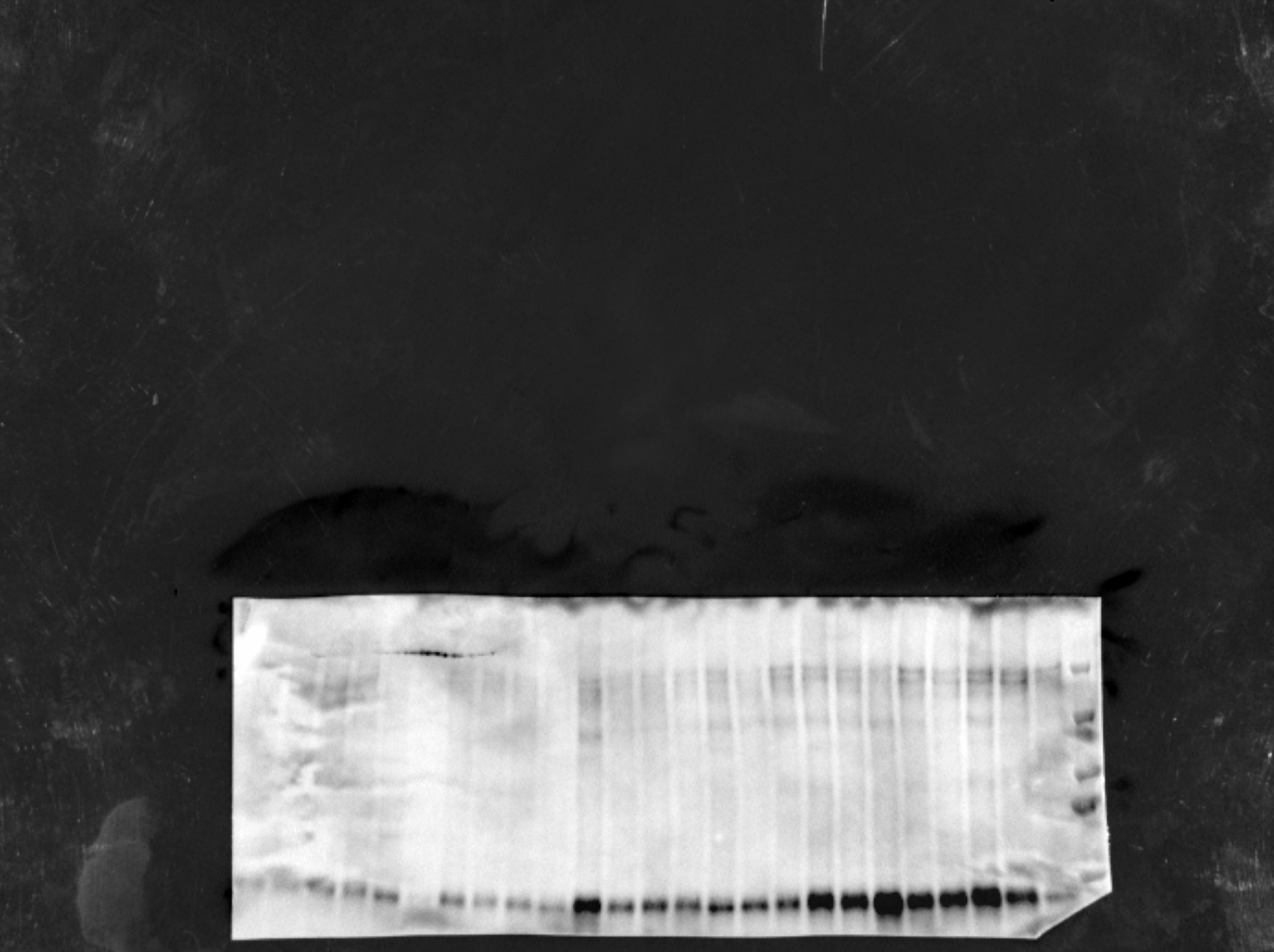

Supplement: Source data 1. [file elife-78387-data1.zip › Western blot source data/raw files/Figure S1 - non-scar 4-sulfation.tiff]

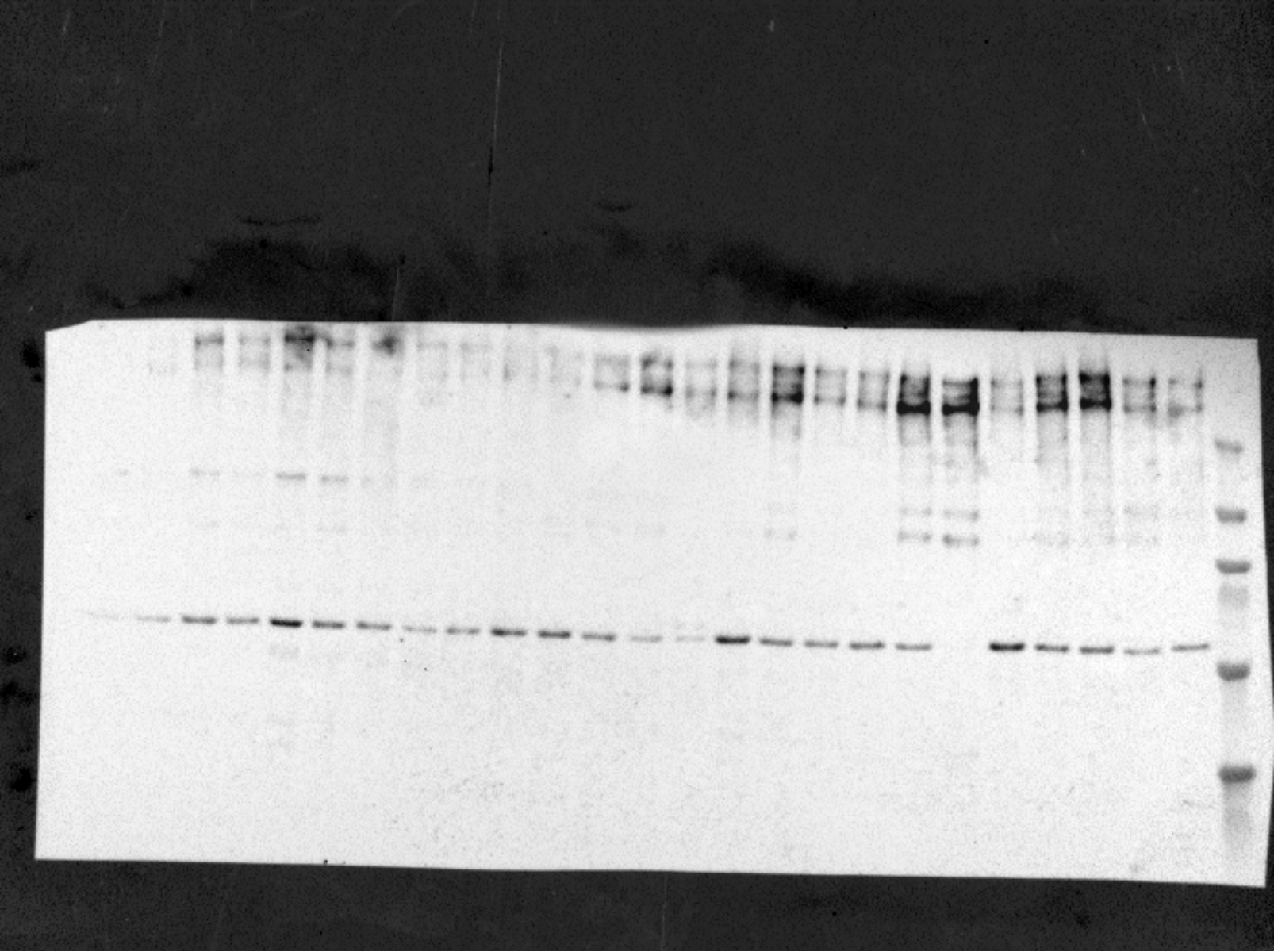

Supplement: Source data 1. [file elife-78387-data1.zip › Western blot source data/raw files/Figure 5 - NG2.tif]

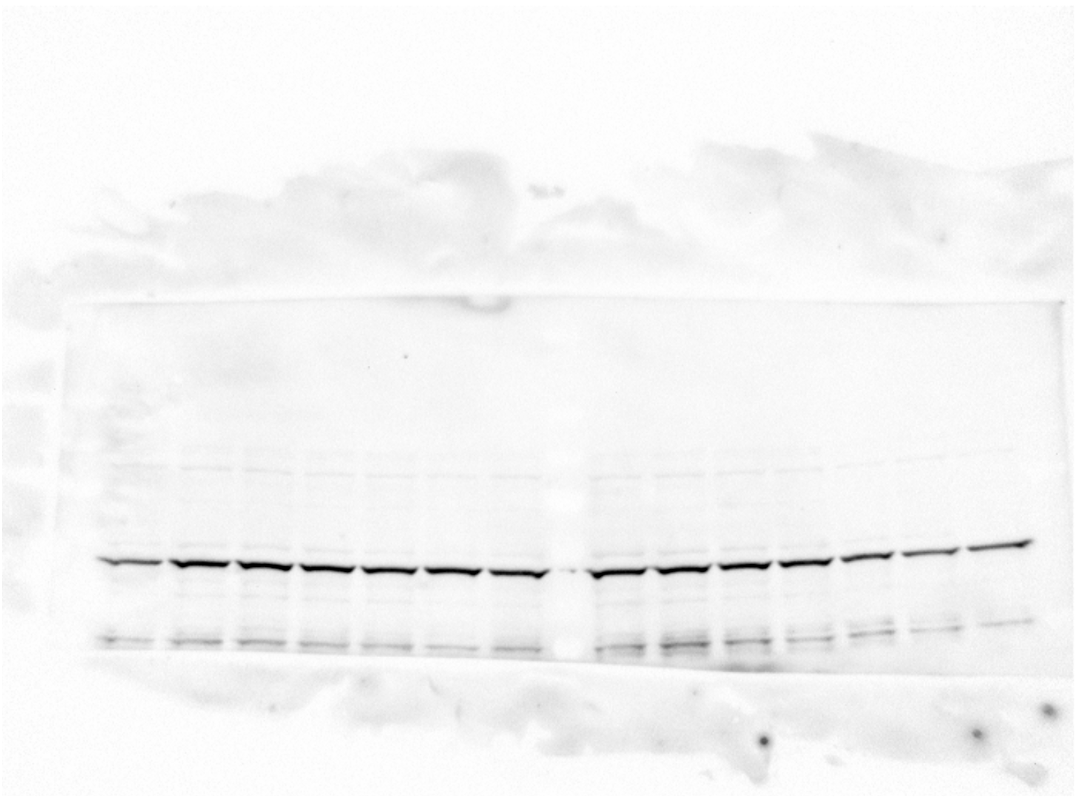

Supplement: Source data 1. [file elife-78387-data1.zip › Western blot source data/raw files/Figure S3 - CHST15.png]

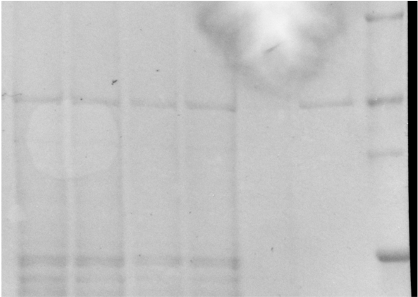

Supplement: Source data 1. [file elife-78387-data1.zip › Western blot source data/raw files/Figure 1 - ponceau.png]

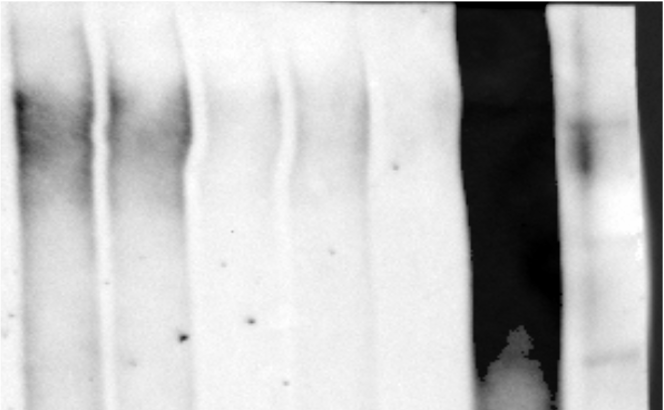

Supplement: Source data 1. [file elife-78387-data1.zip › Western blot source data/raw files/Figure 1 - 6S-CS GAGs.png]

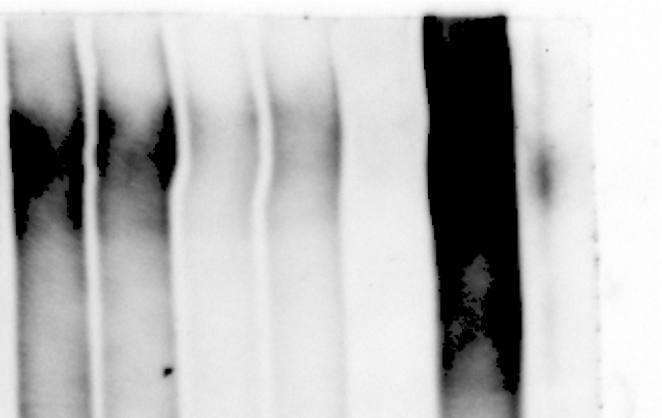

Supplement: Source data 1. [file elife-78387-data1.zip › Western blot source data/raw files/Figure 1 - 4S-CS GAGs.png]

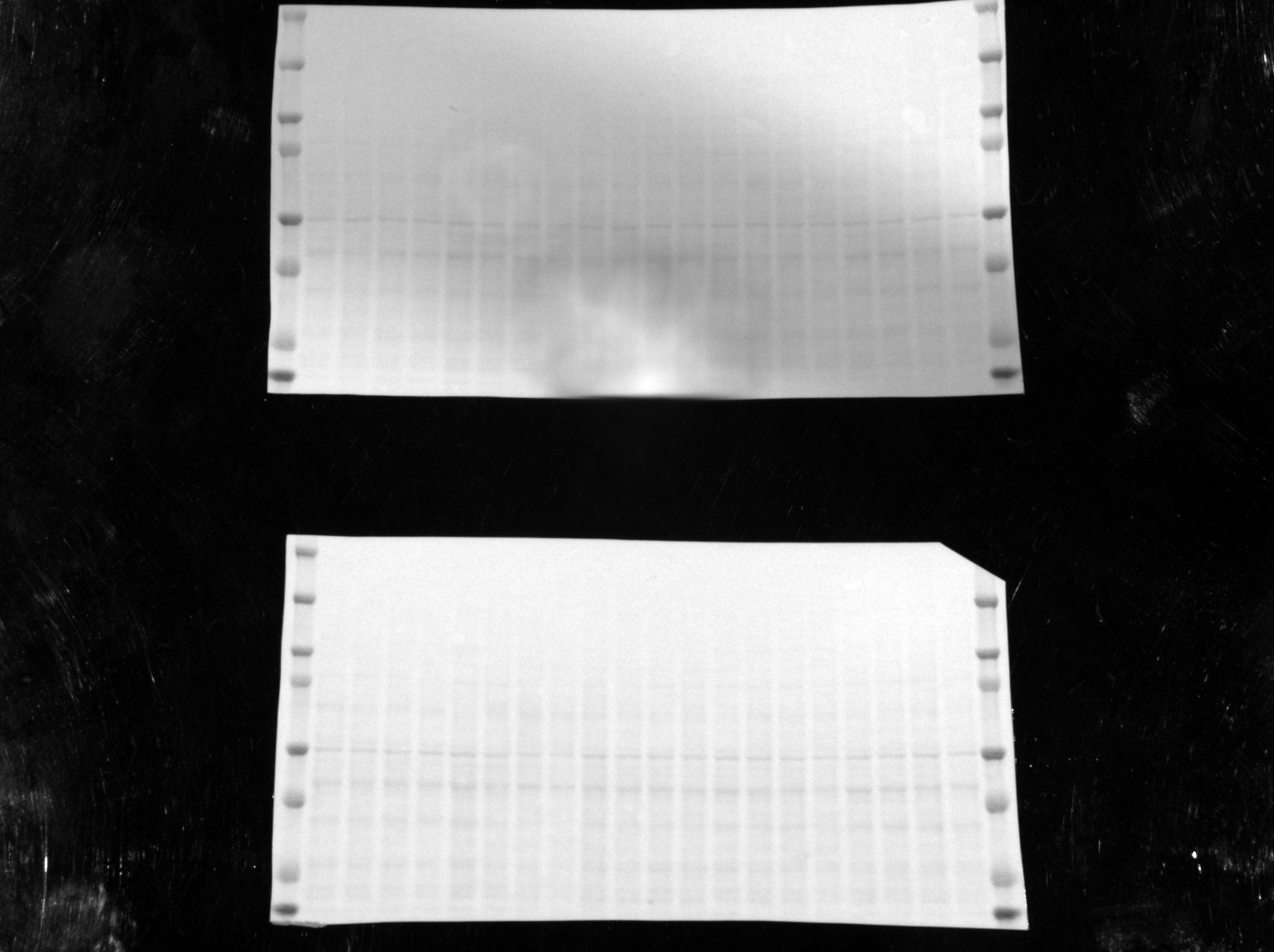

Supplement: Source data 1. [file elife-78387-data1.zip › Western blot source data/raw files/Figure 4 - CHST15 ponceau.tif]

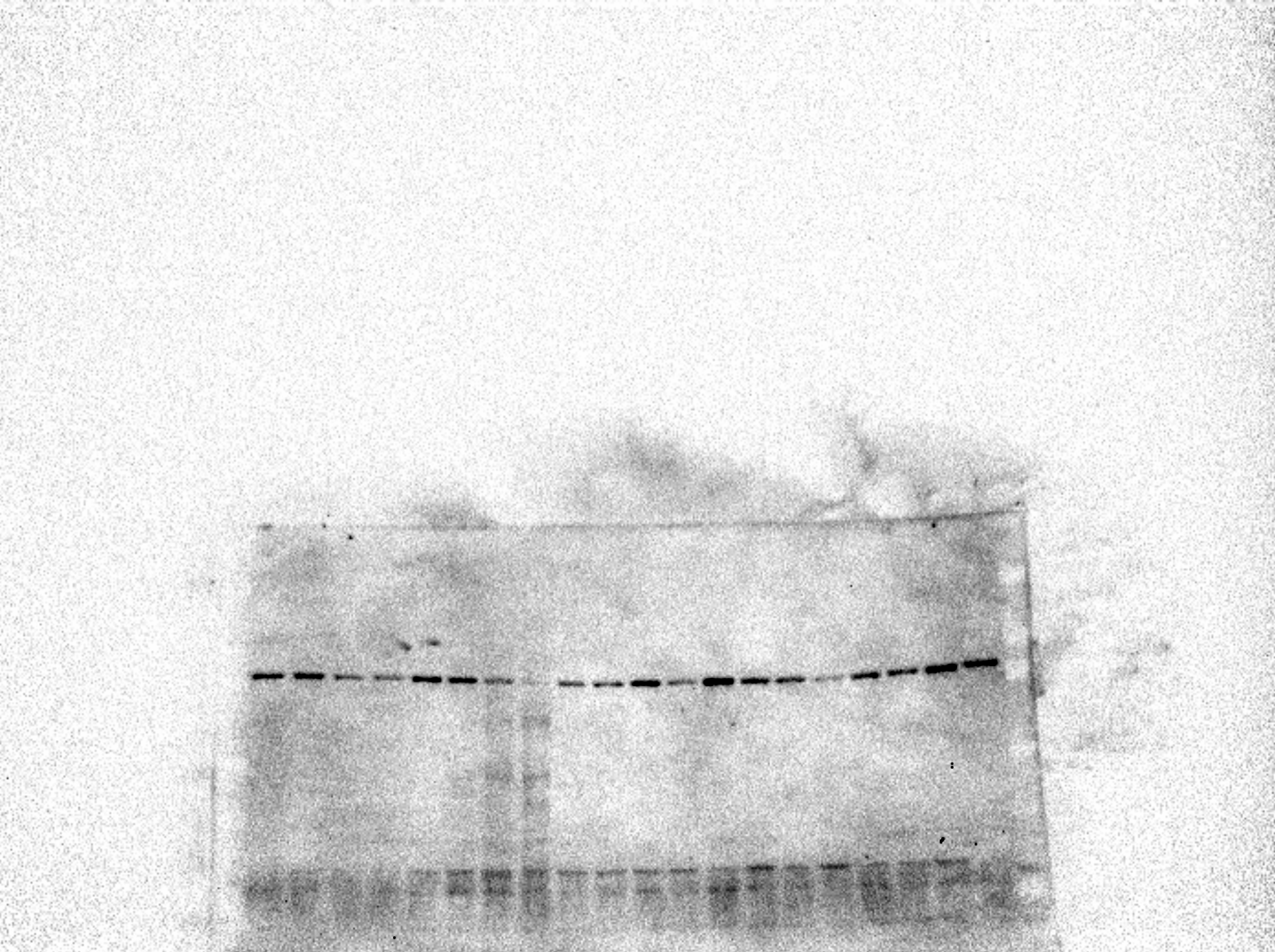

Supplement: Source data 1. [file elife-78387-data1.zip › Western blot source data/raw files/Figure 4 -ponceau.tif]

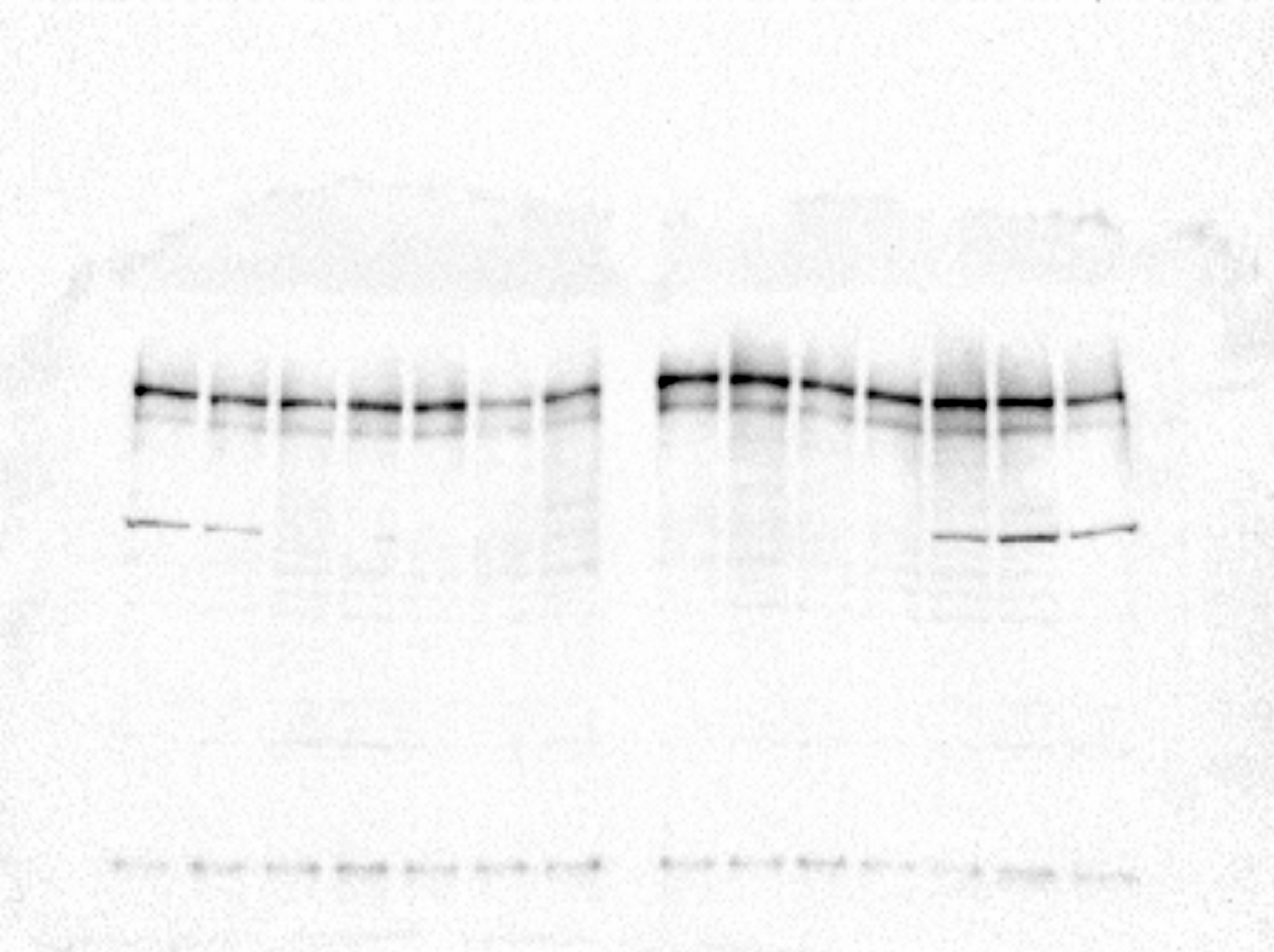

Supplement: Source data 1. [file elife-78387-data1.zip › Western blot source data/raw files/Figure 6 - NG2.tif]

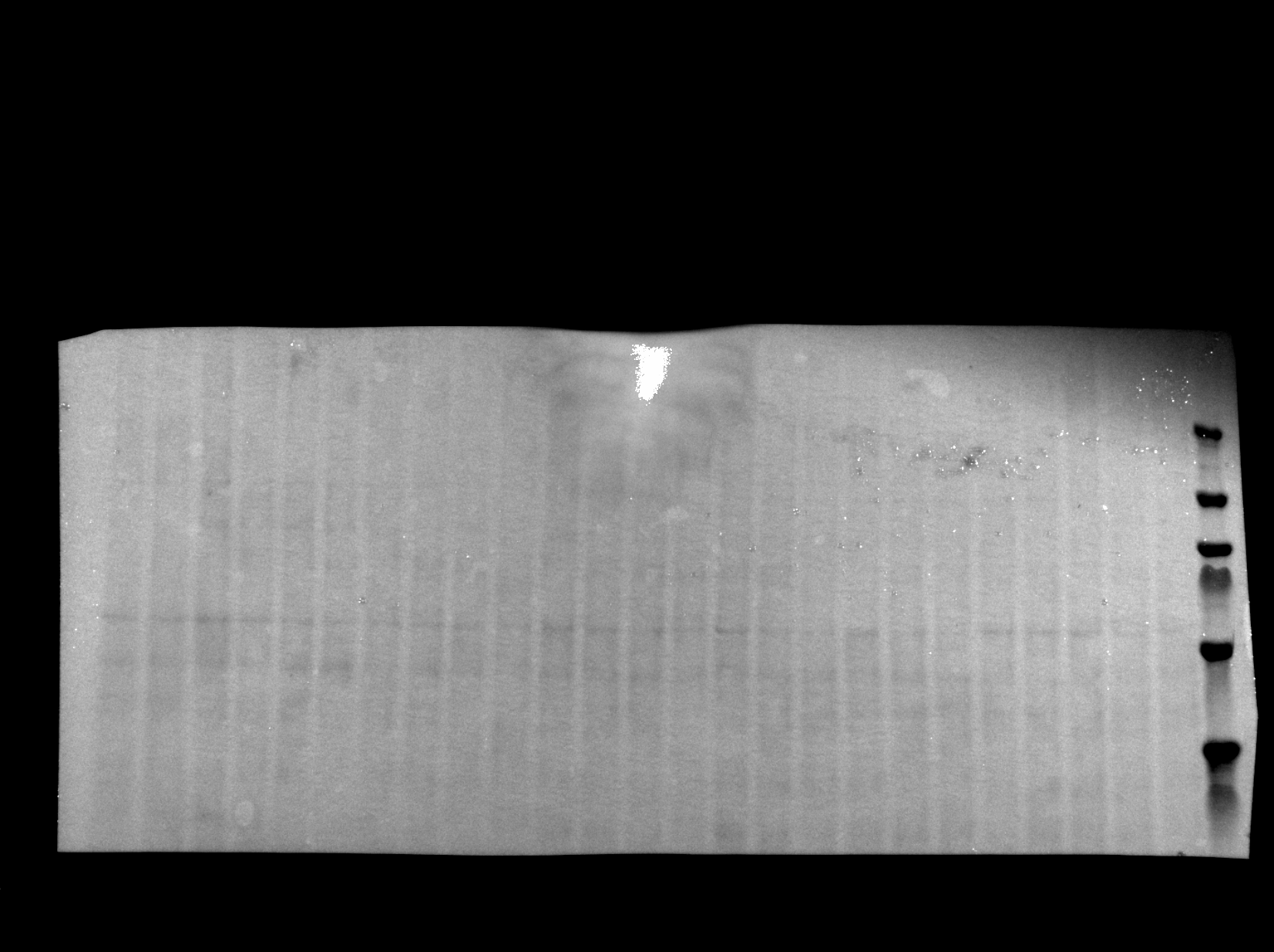

Supplement: Source data 1. [file elife-78387-data1.zip › Western blot source data/raw files/Figure 5 - NG2 ponceau.tif]

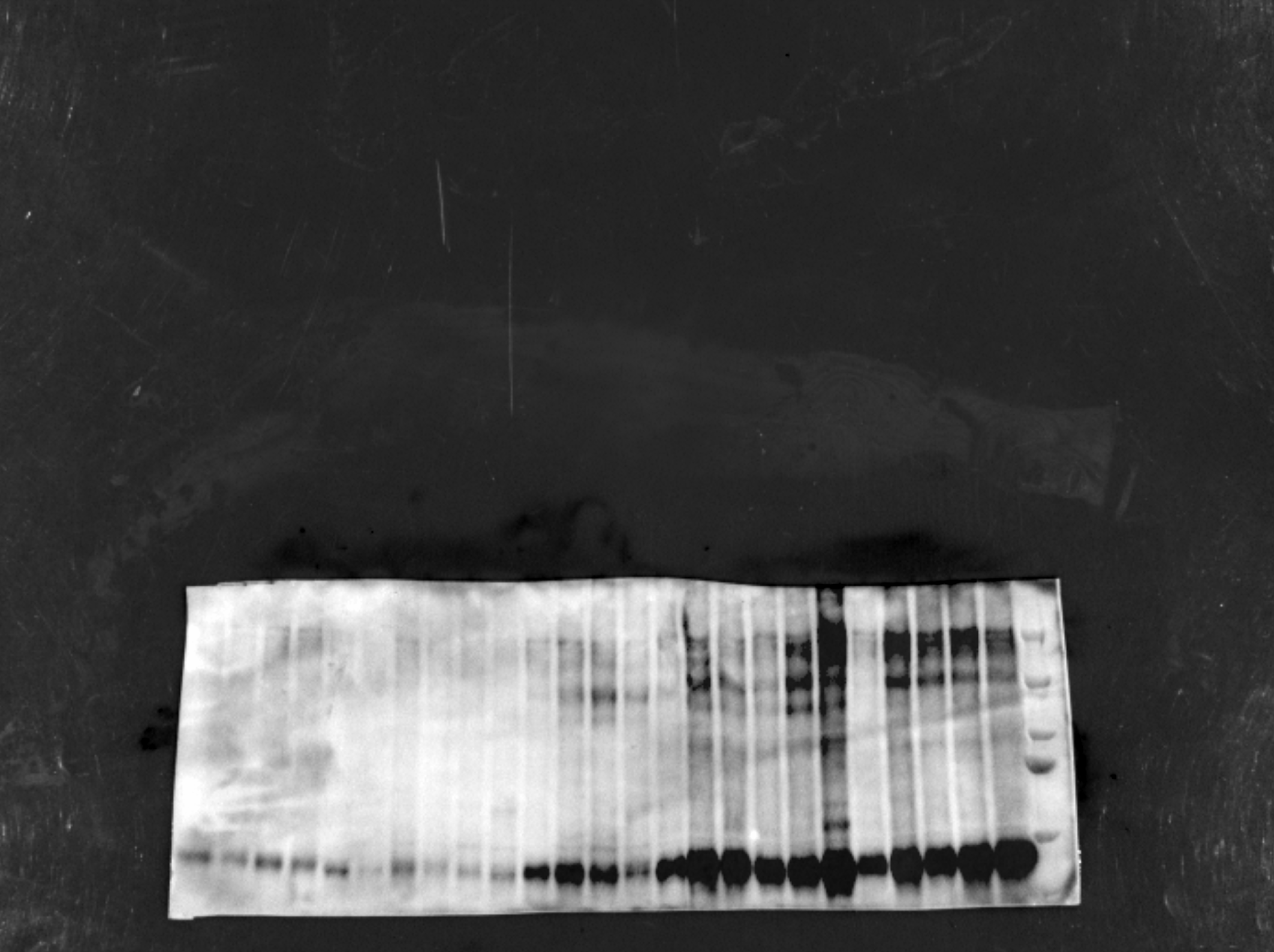

Supplement: Source data 1. [file elife-78387-data1.zip › Western blot source data/raw files/Figure 5 - 4S-CS GAGs.tif]

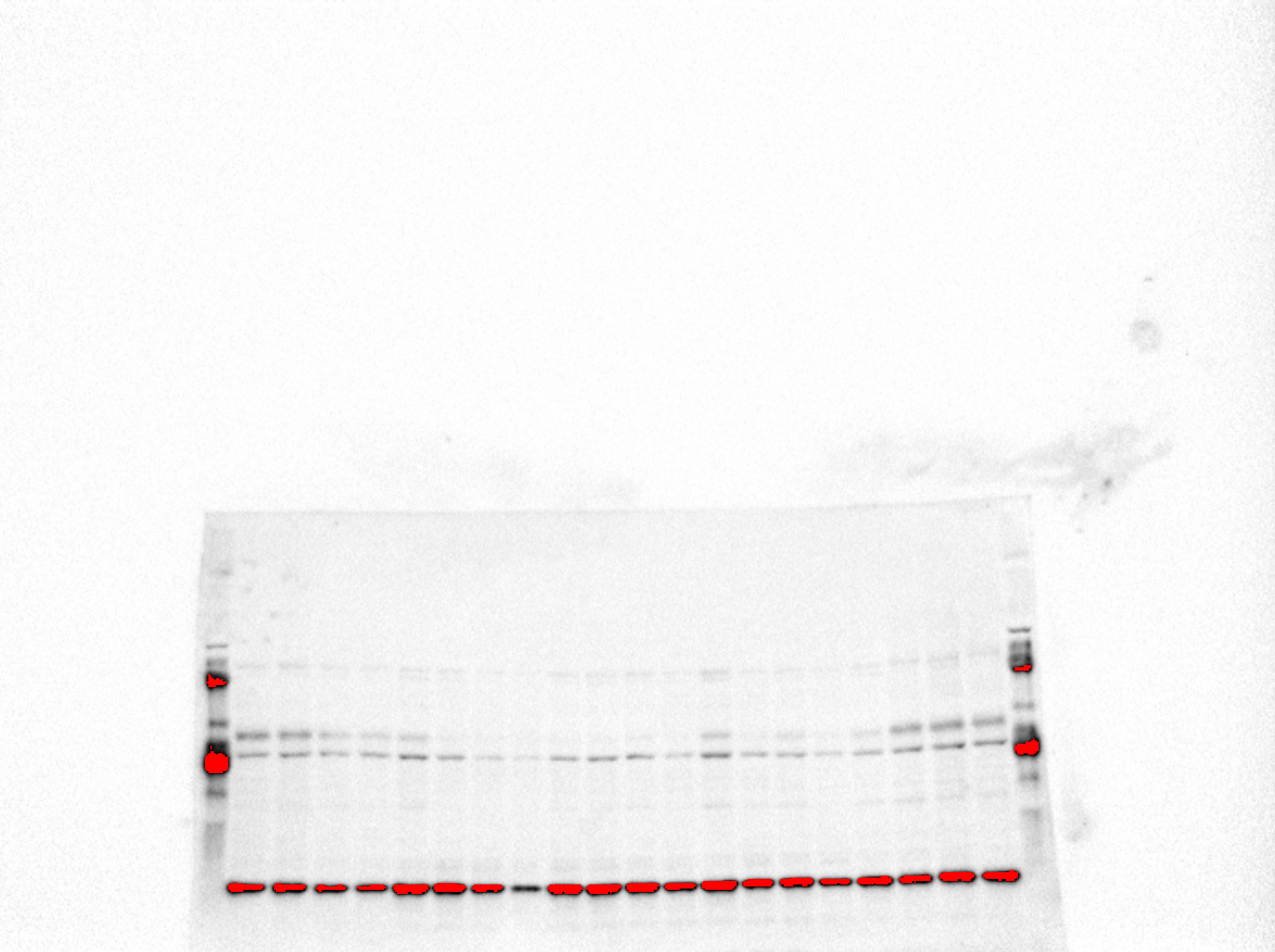

Supplement: Source data 1. [file elife-78387-data1.zip › Western blot source data/raw files/Figure 4 ARSB.tif]

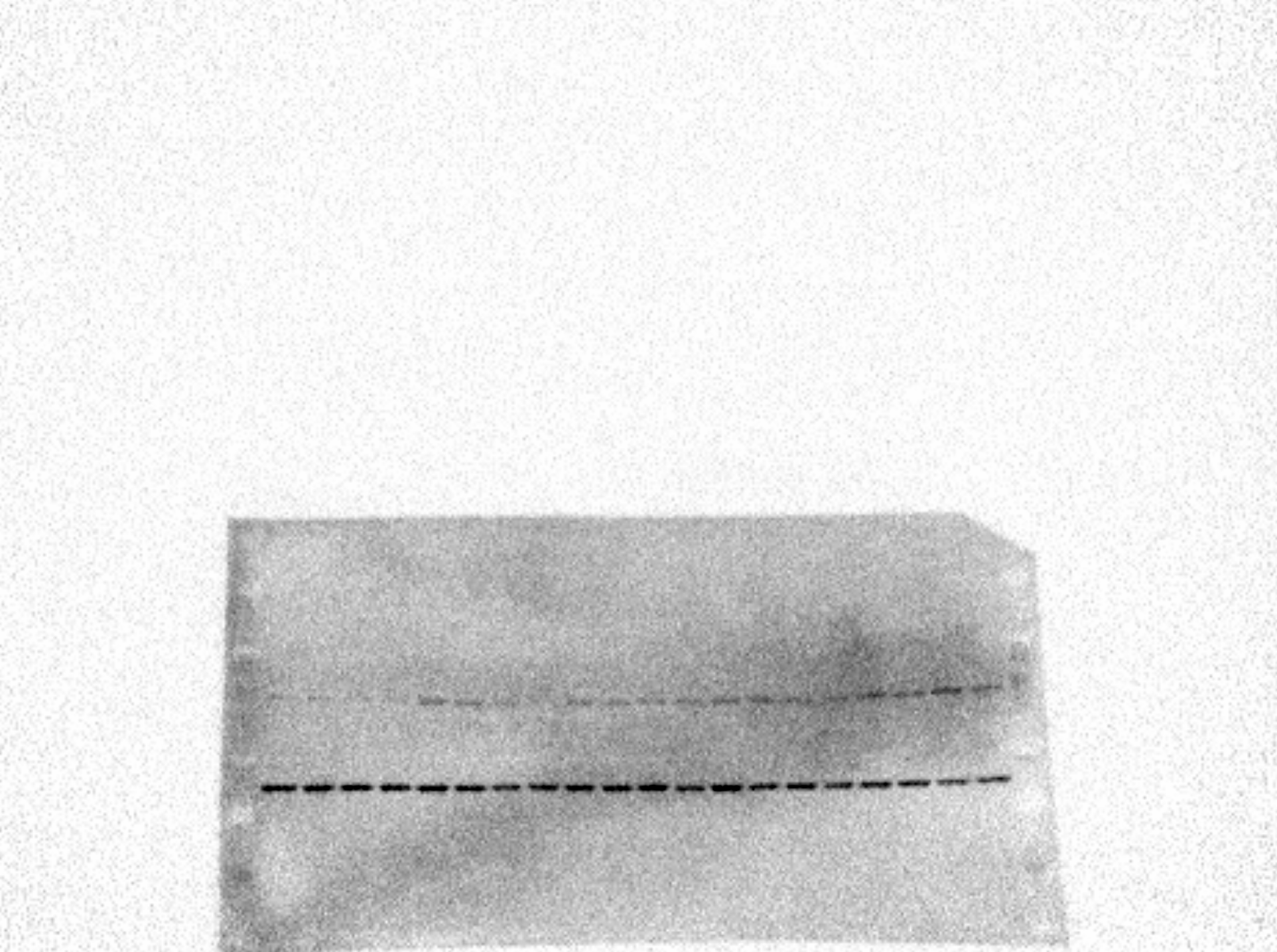

Supplement: Source data 1. [file elife-78387-data1.zip › Western blot source data/raw files/Figure 4 - CHST15.tif]

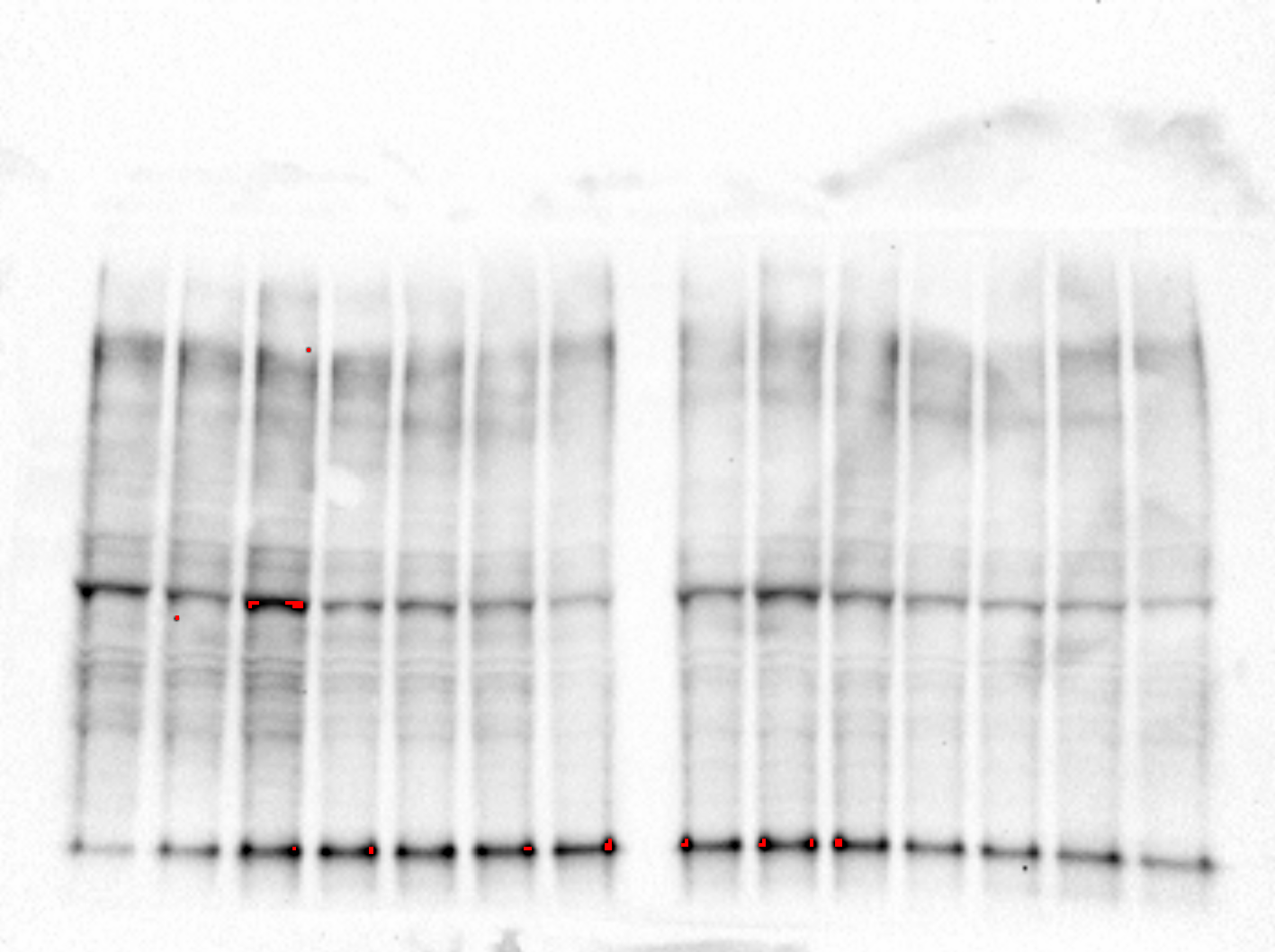

Supplement: Source data 1. [file elife-78387-data1.zip › Western blot source data/raw files/Figure 6 - 6S-CS GAGs.tif]

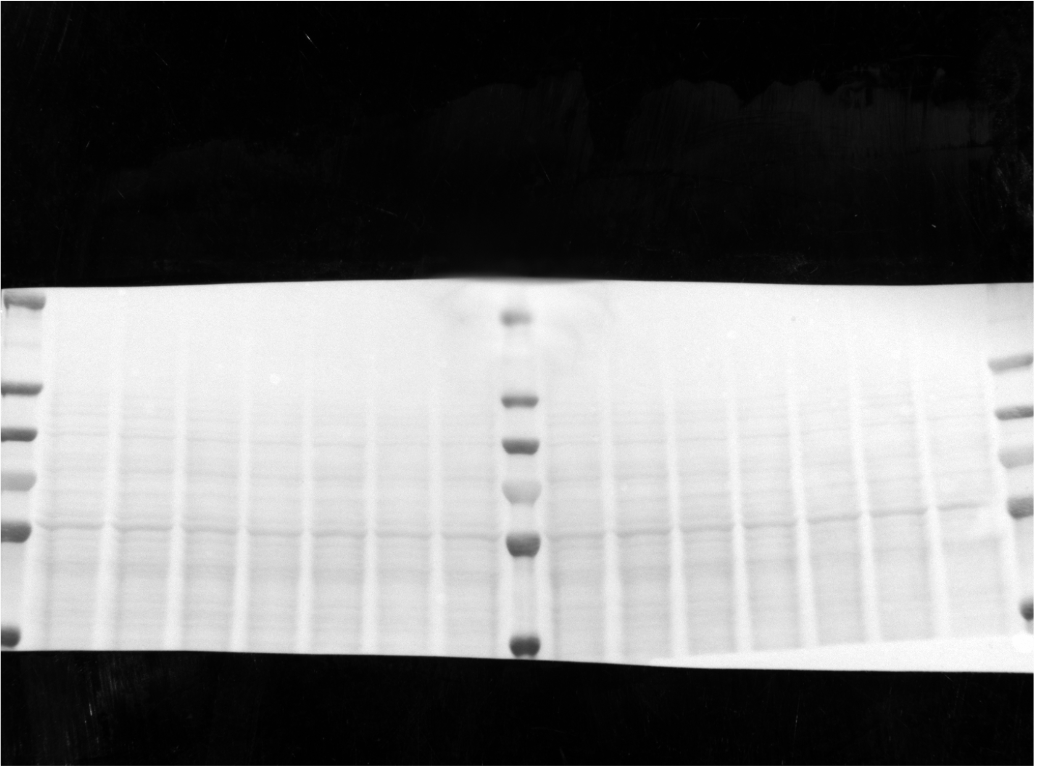

Supplement: Source data 1. [file elife-78387-data1.zip › Western blot source data/raw files/Figure S3- ponceau CHST15.png]

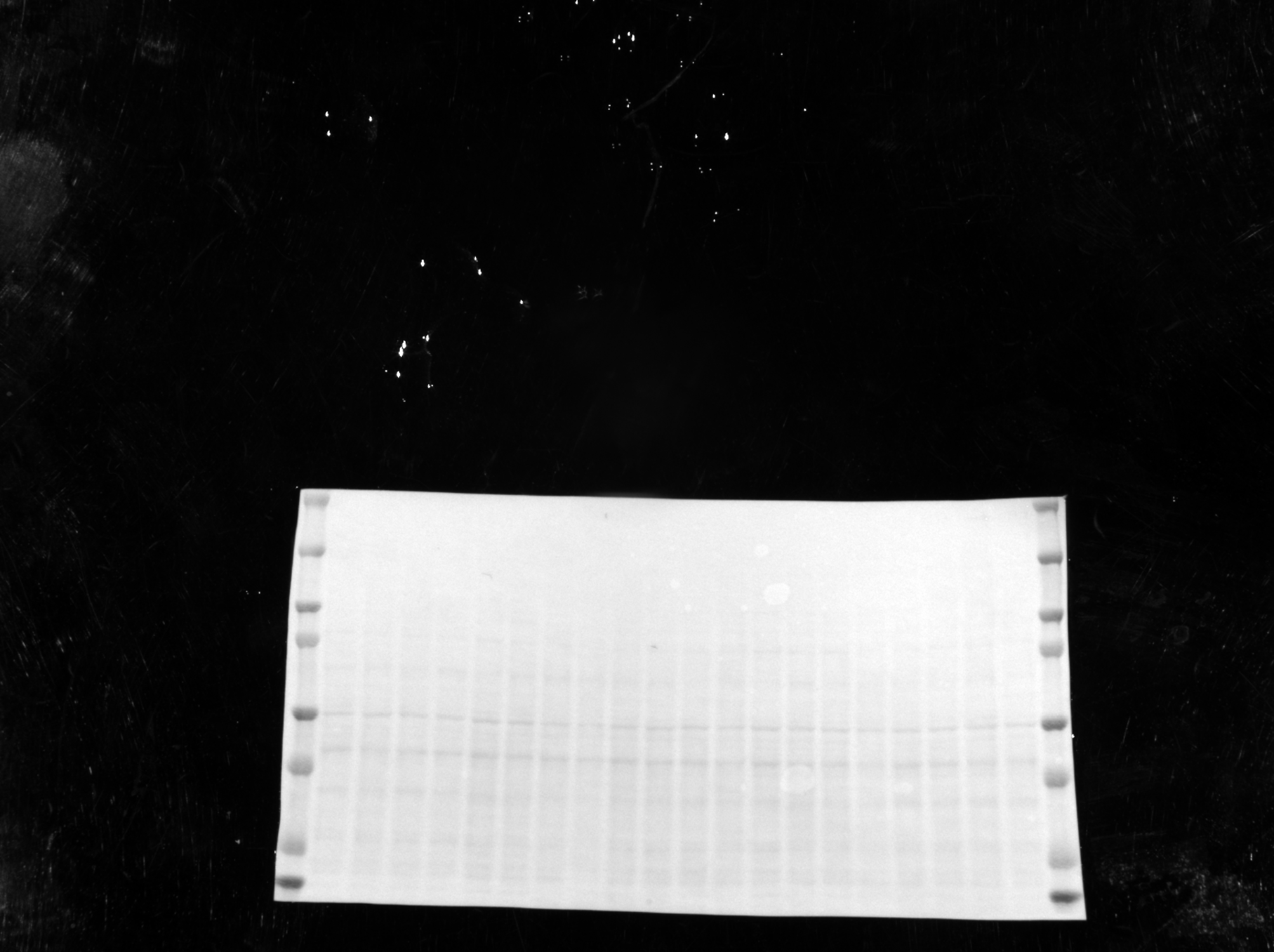

Supplement: Source data 1. [file elife-78387-data1.zip › Western blot source data/raw files/Figure 4 - Ponceau.tif]

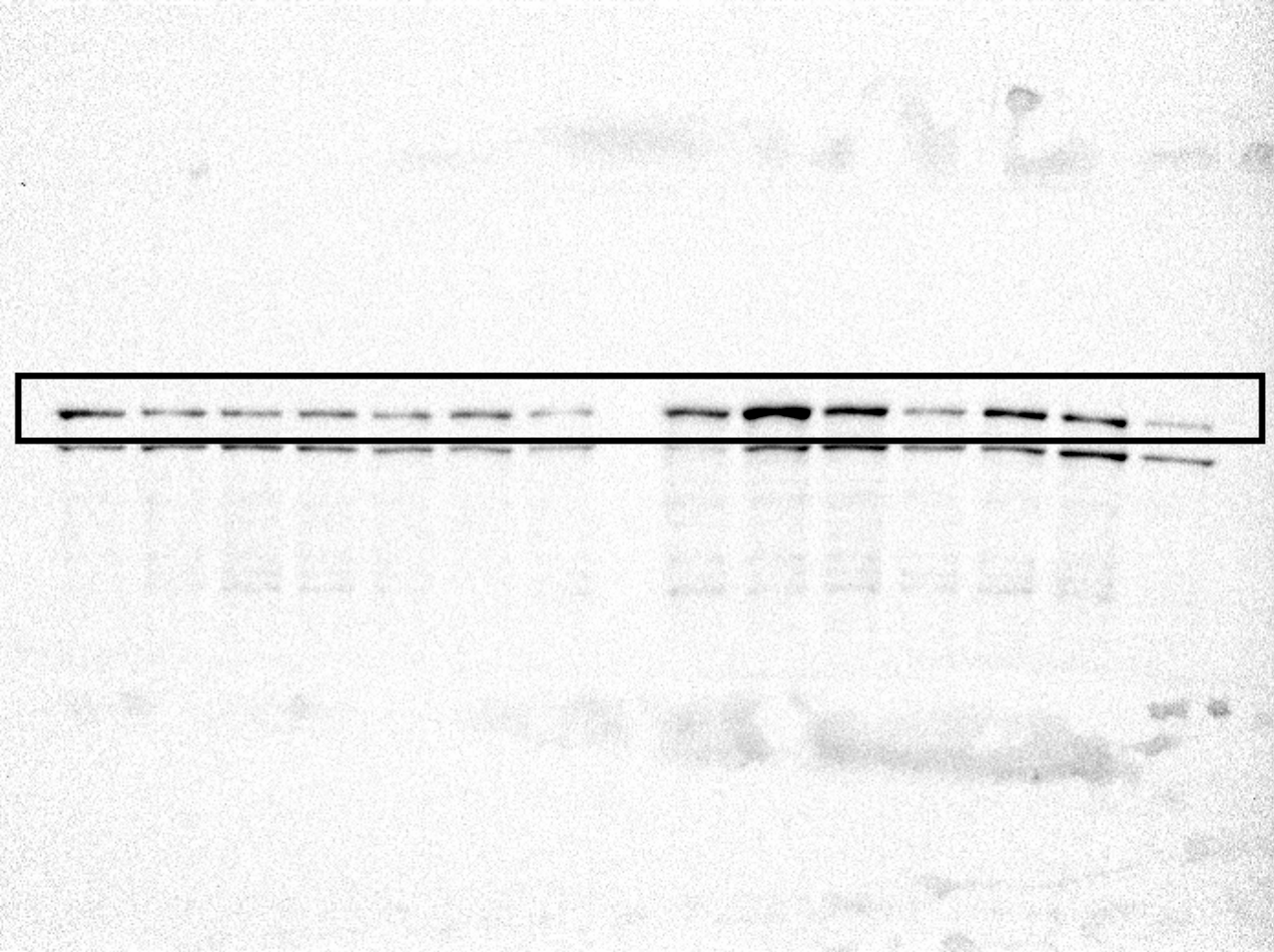

Supplement: Source data 1. [file elife-78387-data1.zip › Western blot source data/WB band outlined/Figure 6 - TH.tif]

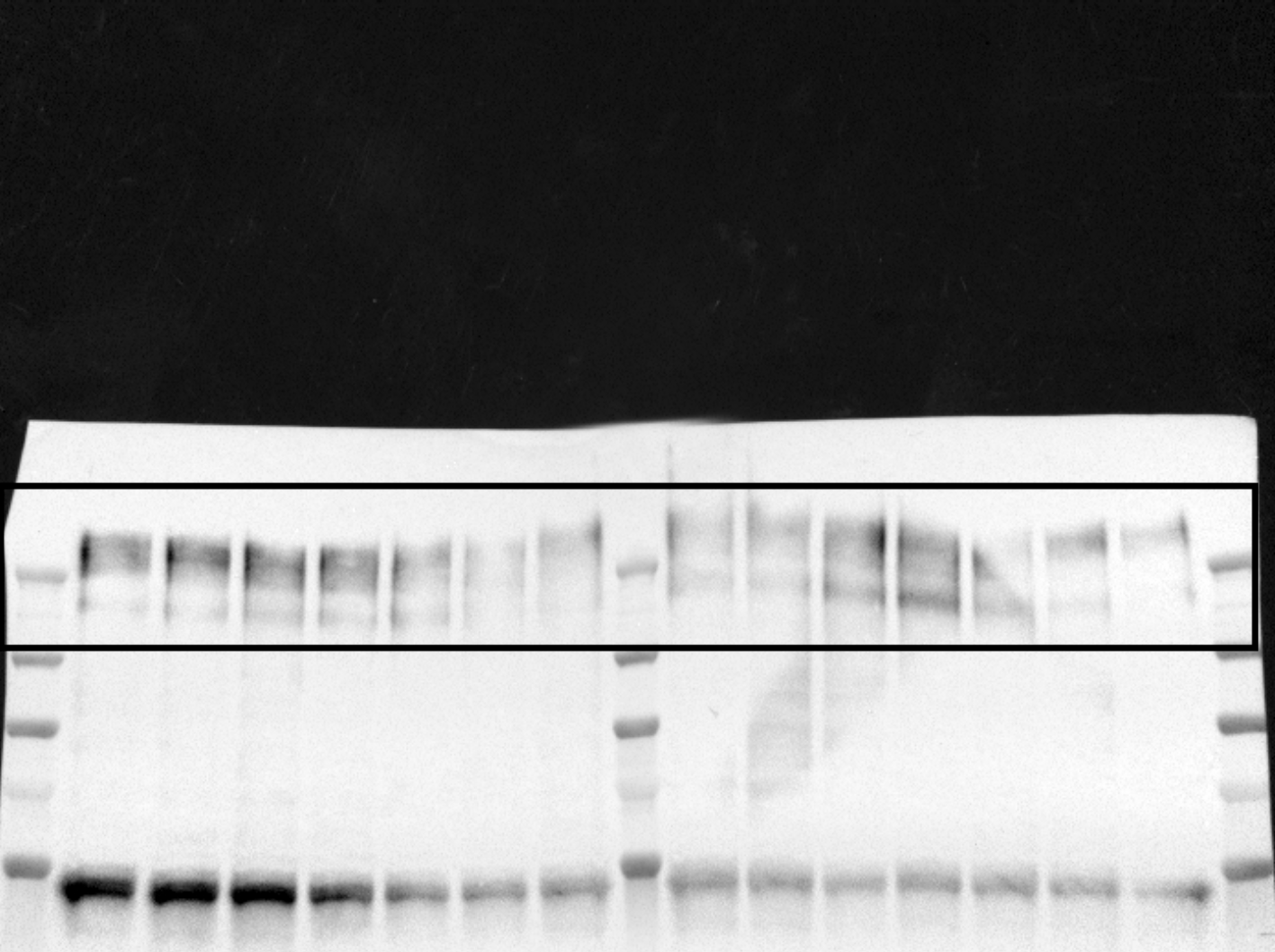

Supplement: Source data 1. [file elife-78387-data1.zip › Western blot source data/WB band outlined/Figure 6 - 4S-CS GAGs.tif]

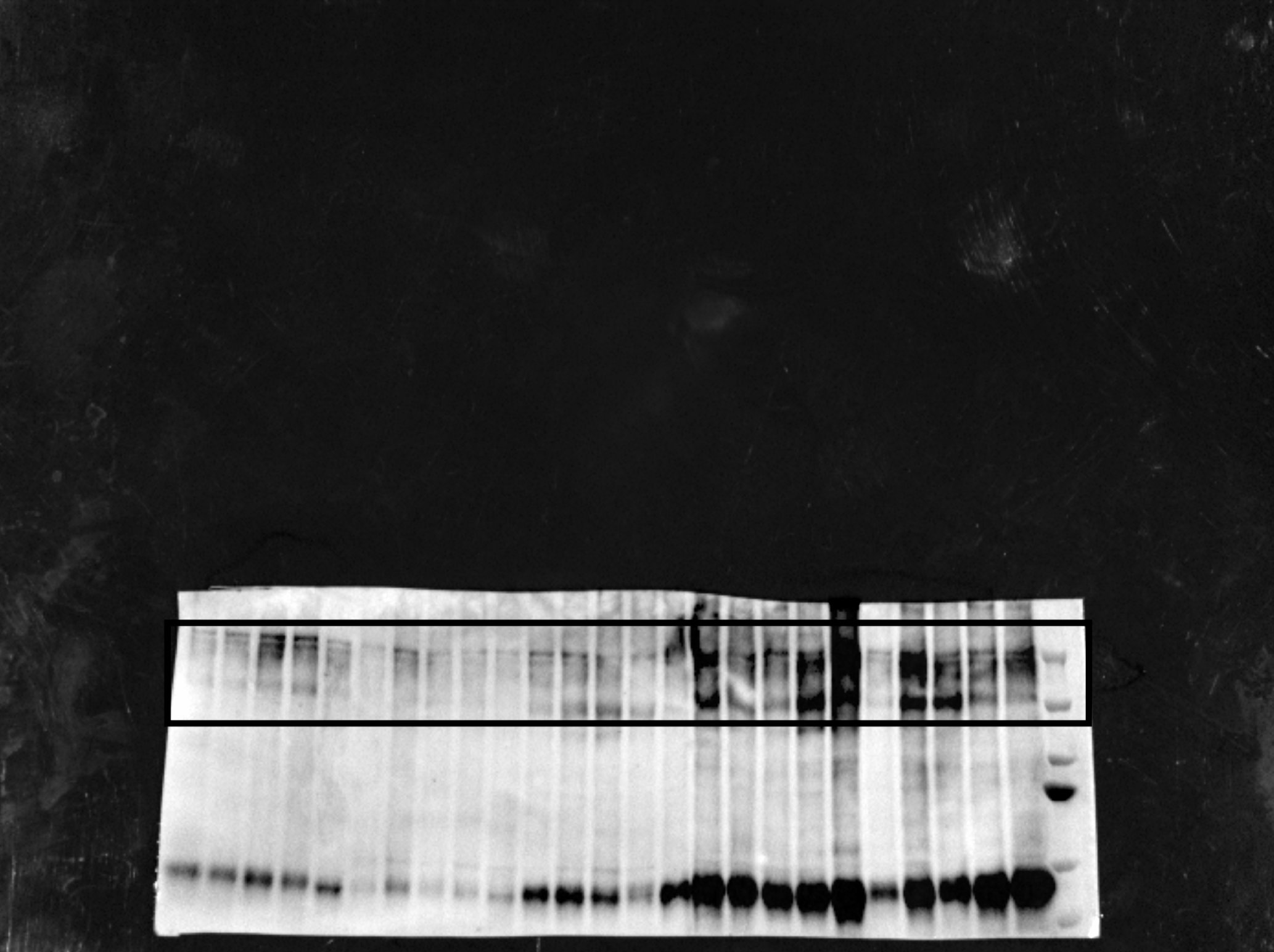

Supplement: Source data 1. [file elife-78387-data1.zip › Western blot source data/WB band outlined/Figure 5 - 6S-CS GAGs.tif]

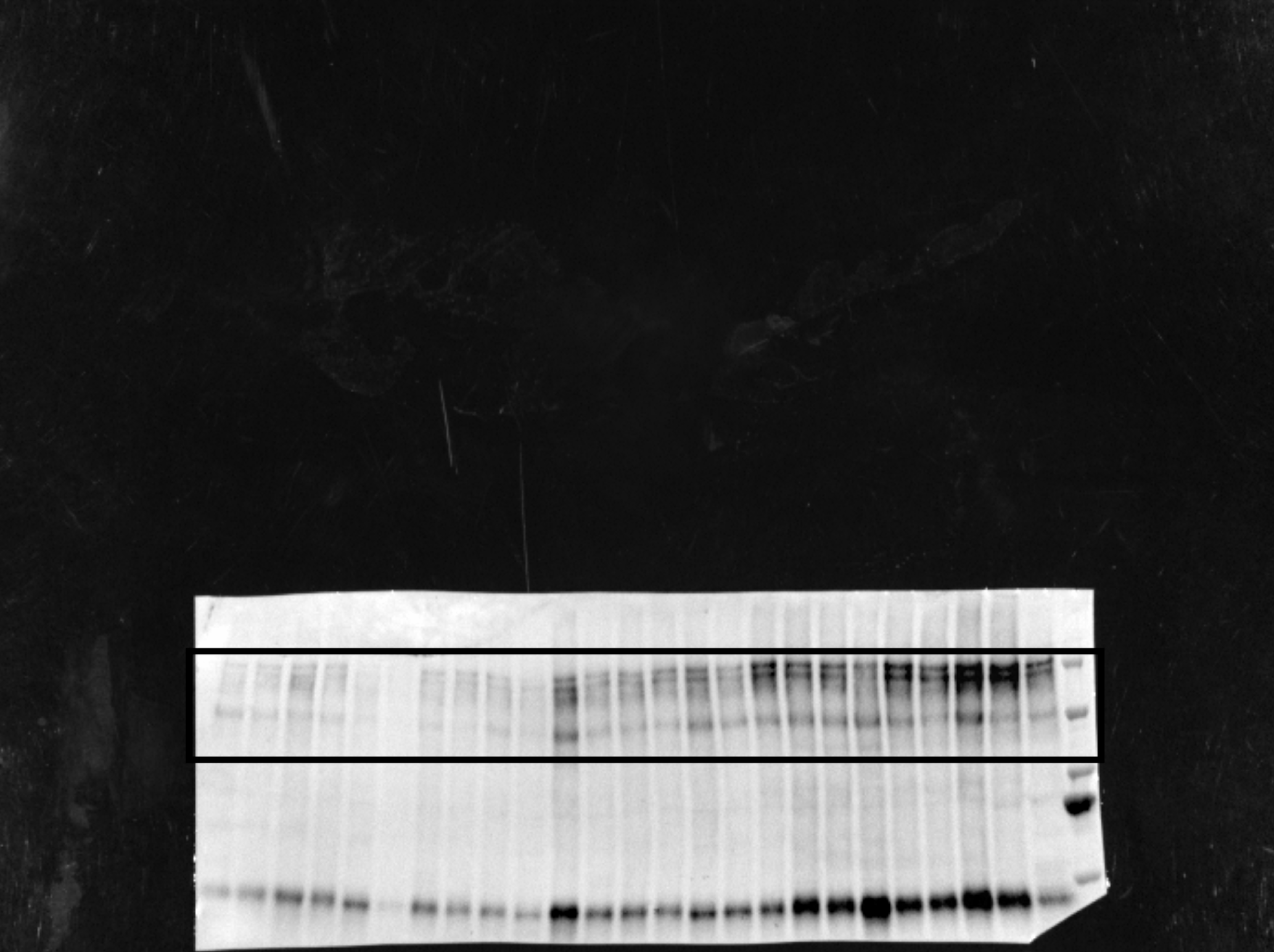

Supplement: Source data 1. [file elife-78387-data1.zip › Western blot source data/WB band outlined/Figure S1 - non-scar 6-sulfation.tiff]

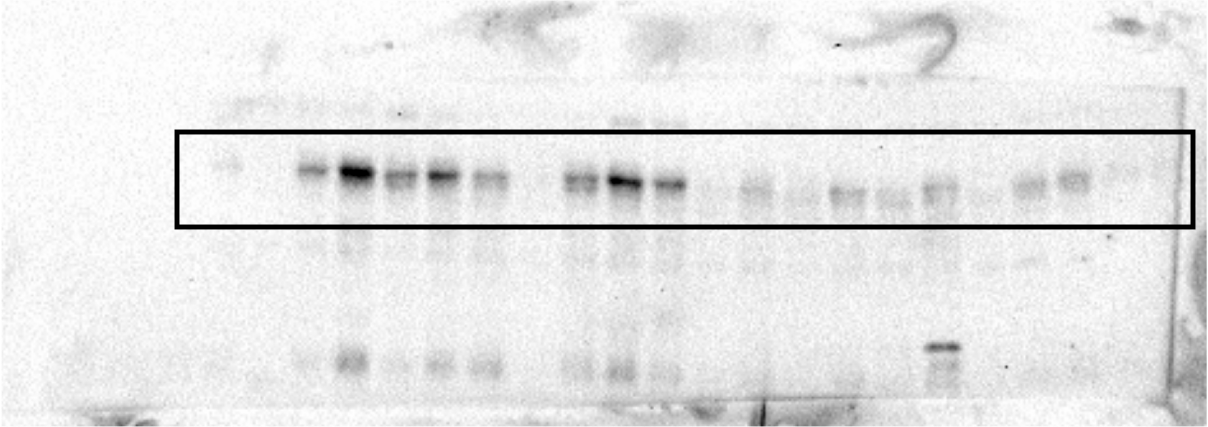

Supplement: Source data 1. [file elife-78387-data1.zip › Western blot source data/WB band outlined/Figure 5 - Galectin-3.png]

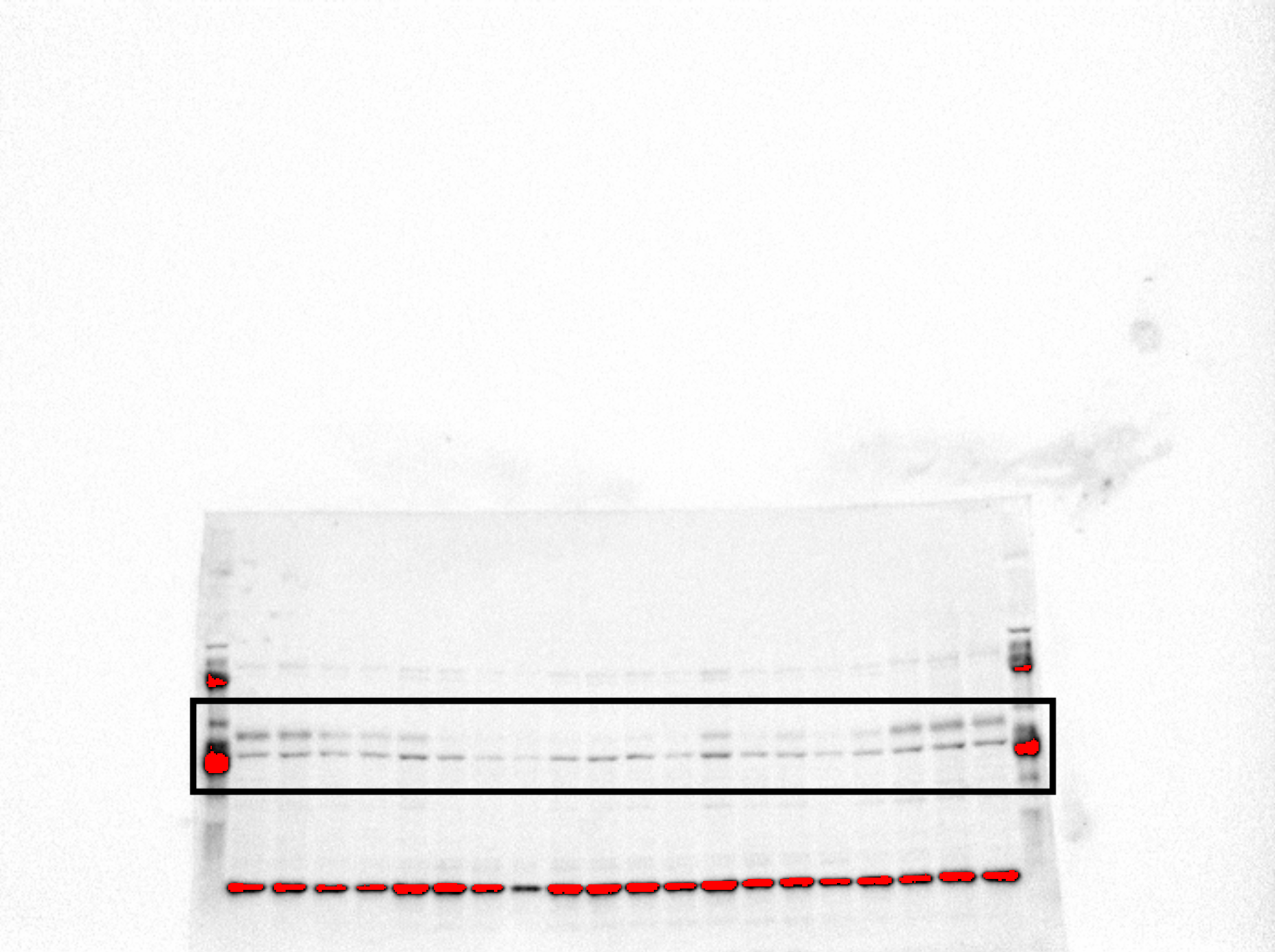

Supplement: Source data 1. [file elife-78387-data1.zip › Western blot source data/WB band outlined/Figure 4 - ARSB.tif]

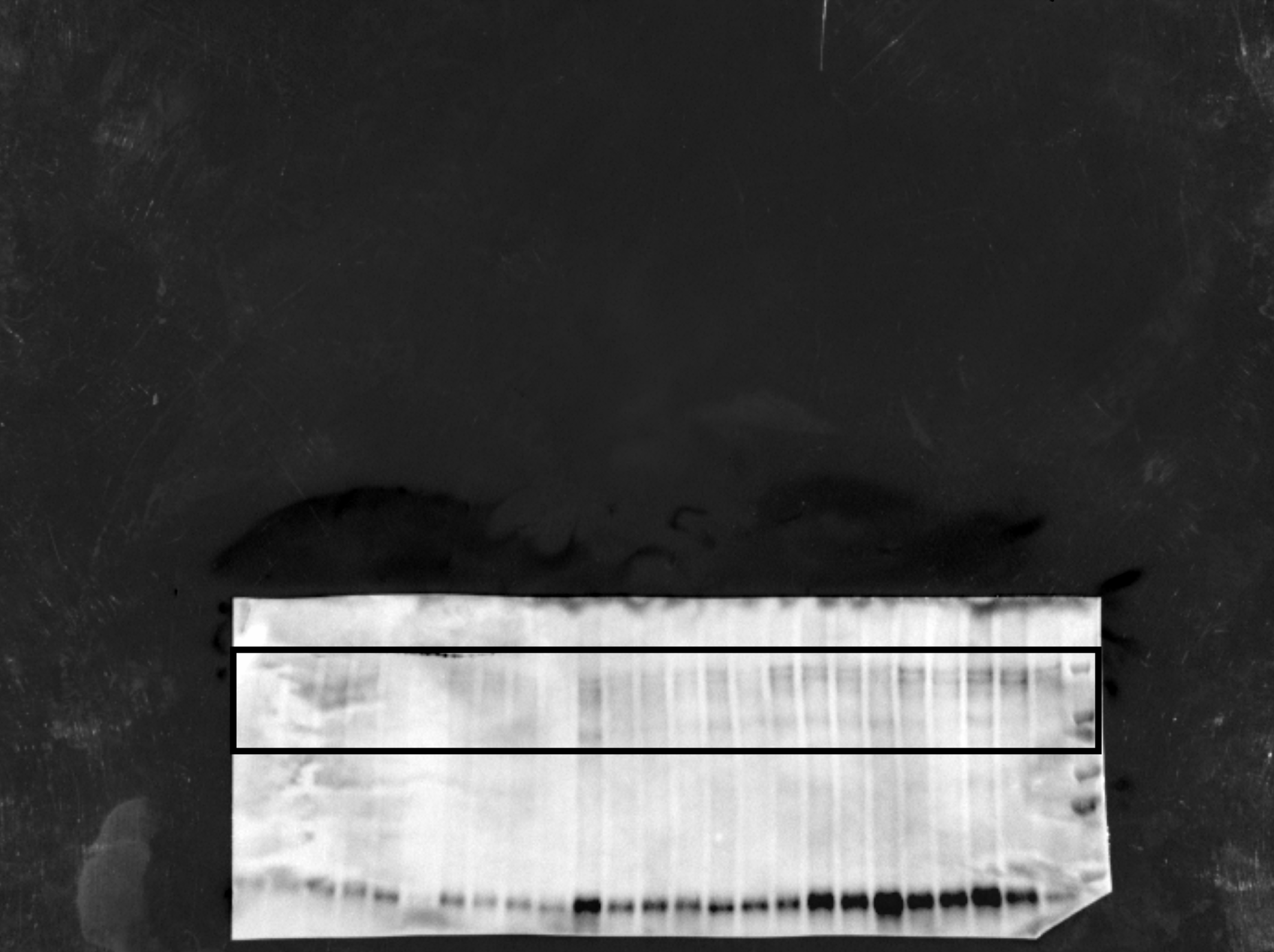

Supplement: Source data 1. [file elife-78387-data1.zip › Western blot source data/WB band outlined/Figure S1 - non-scar 4-sulfation.tiff]

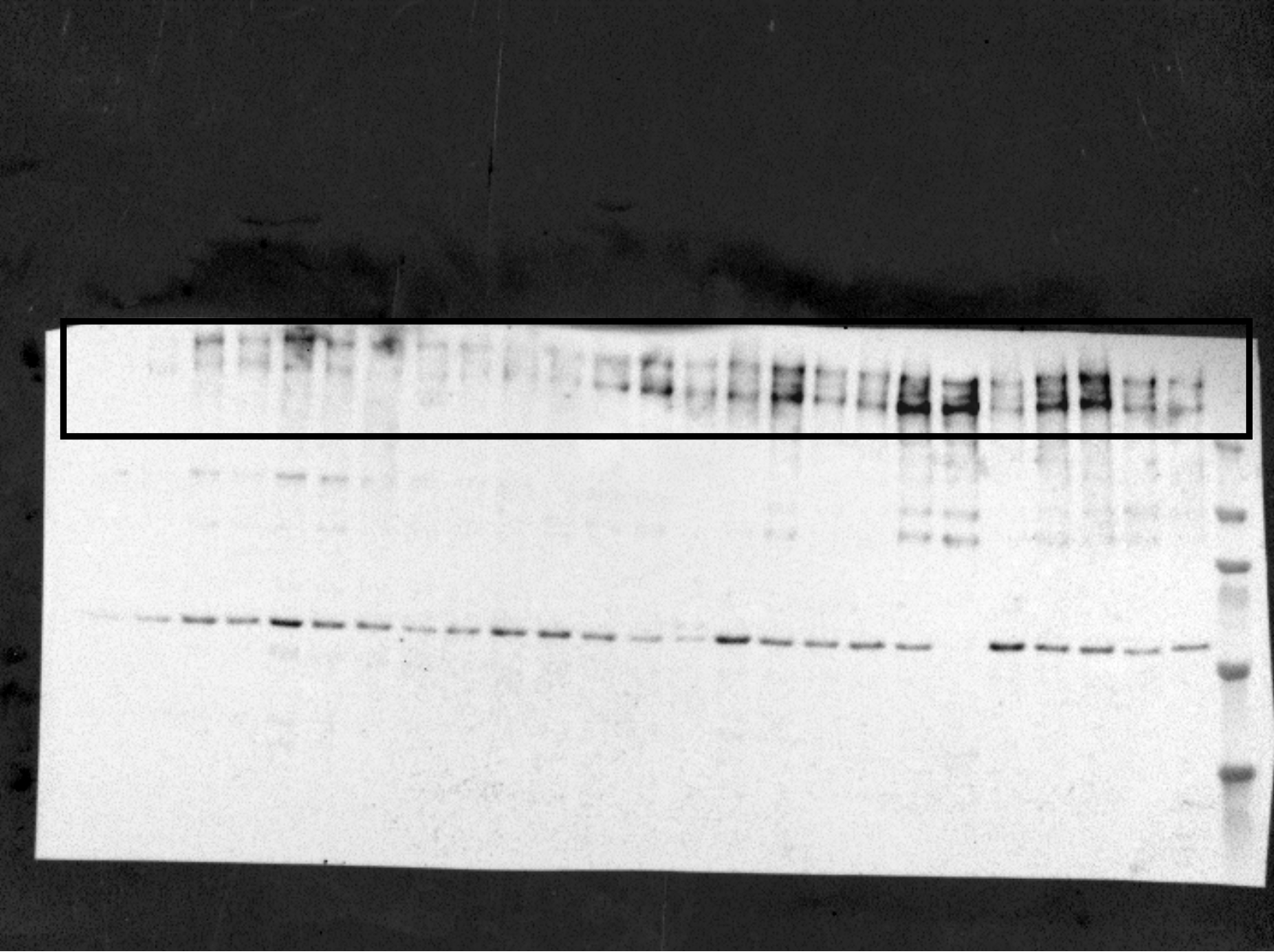

Supplement: Source data 1. [file elife-78387-data1.zip › Western blot source data/WB band outlined/Figure 5 - NG2.tif]

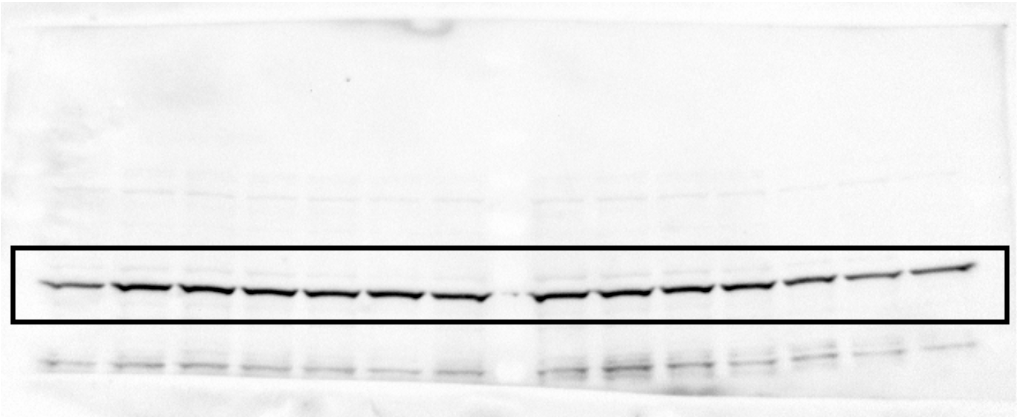

Supplement: Source data 1. [file elife-78387-data1.zip › Western blot source data/WB band outlined/Figure S3 - CHST15.png]

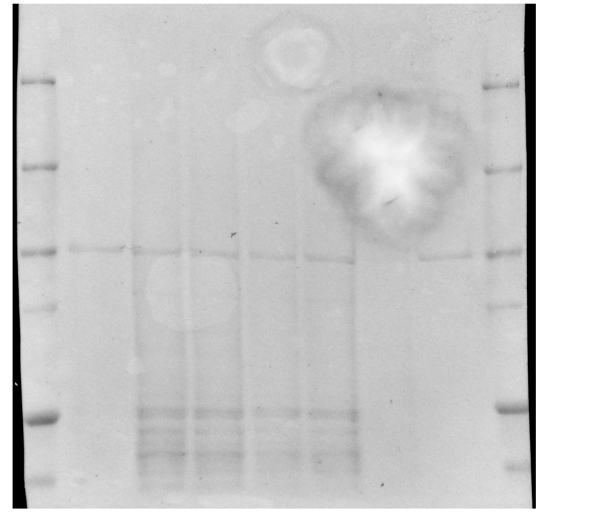

Supplement: Source data 1. [file elife-78387-data1.zip › Western blot source data/WB band outlined/Figure 1 - ponceau.png]

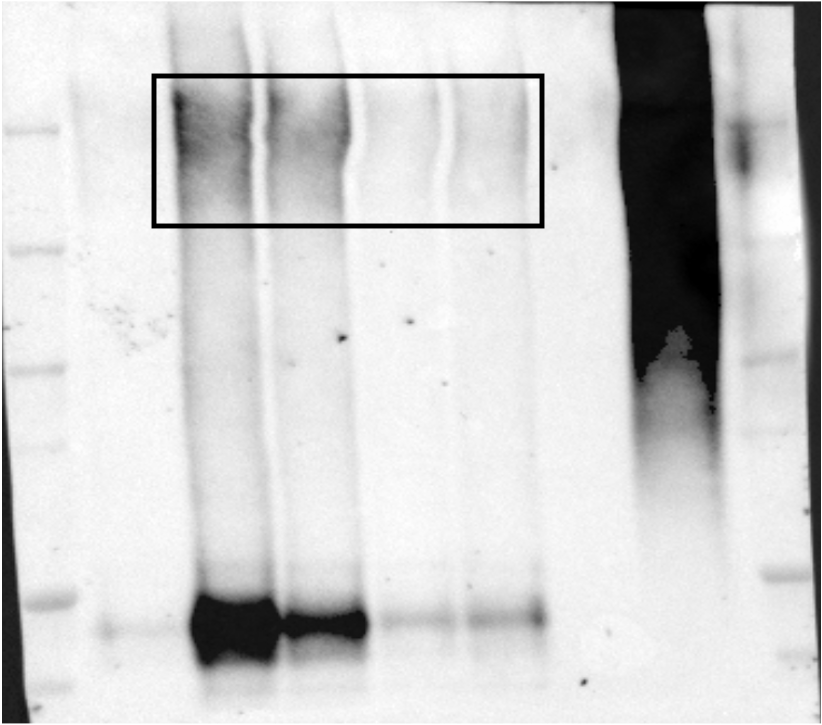

Supplement: Source data 1. [file elife-78387-data1.zip › Western blot source data/WB band outlined/Figure 1 - 6S-CS GAG.png]

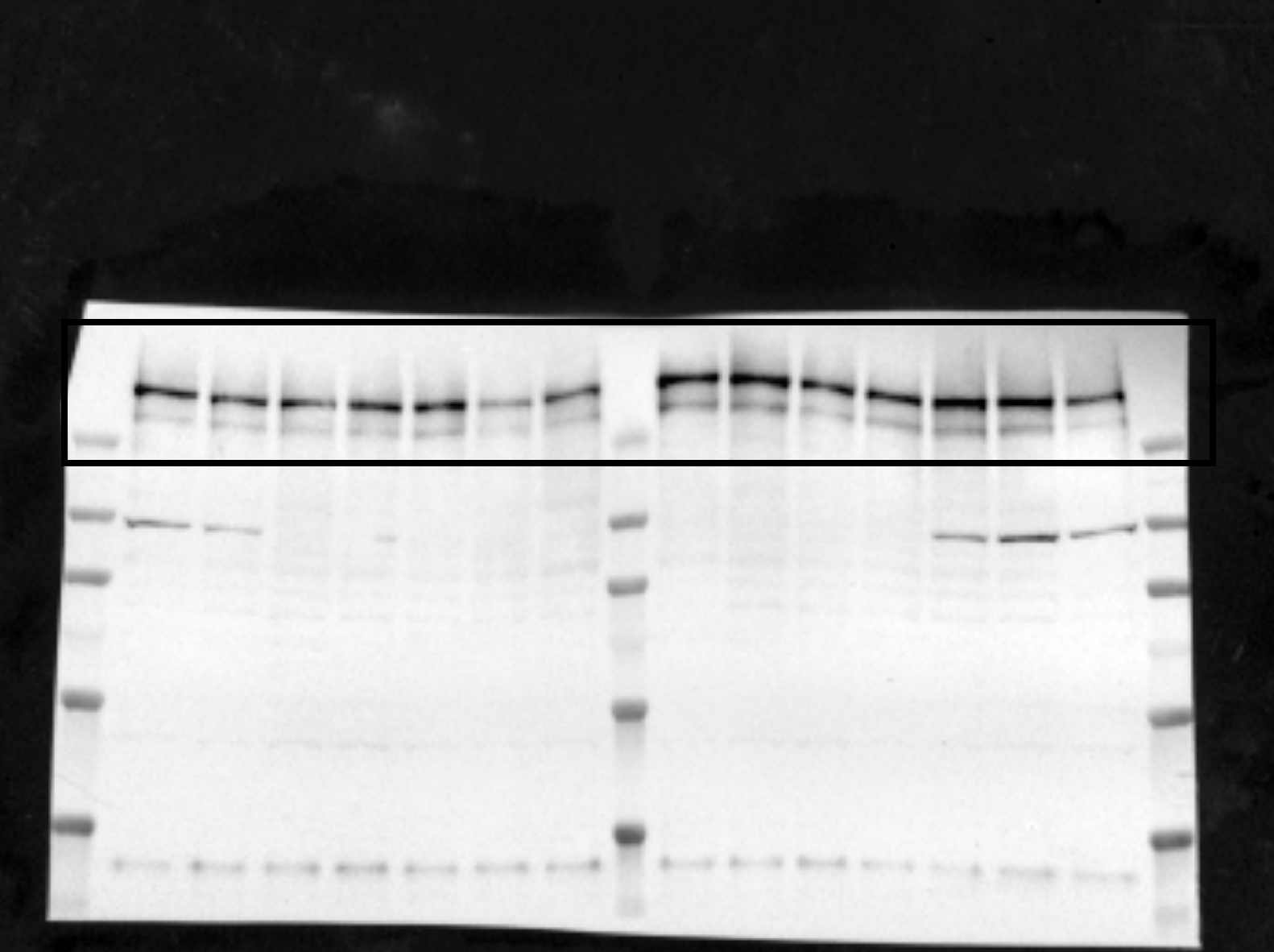

Supplement: Source data 1. [file elife-78387-data1.zip › Western blot source data/WB band outlined/Figure 6 - NG2.tif]

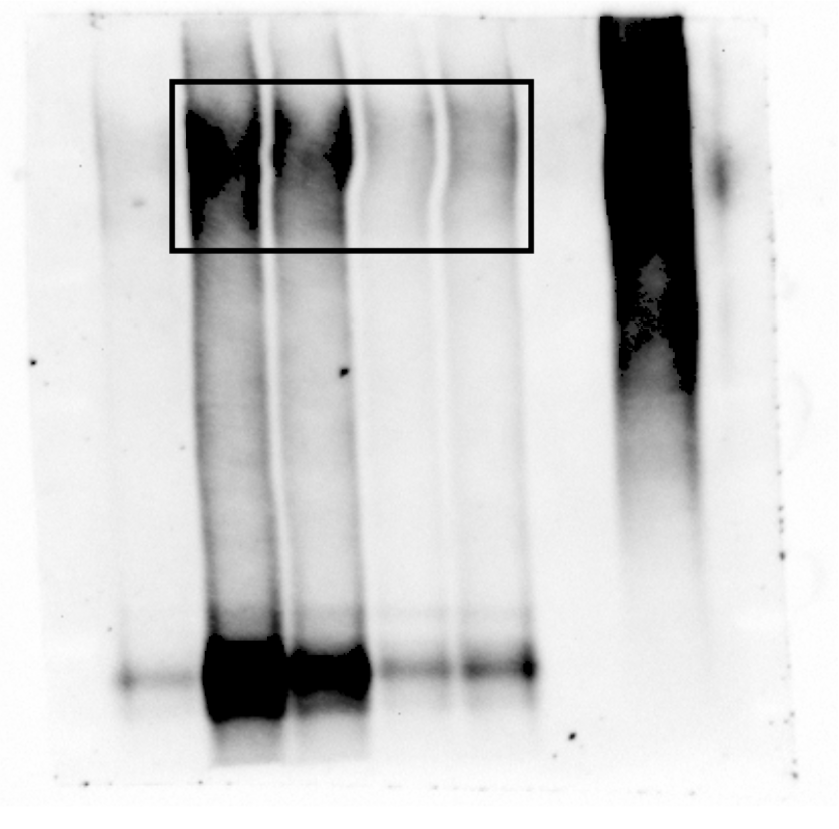

Supplement: Source data 1. [file elife-78387-data1.zip › Western blot source data/WB band outlined/Figure 1 - 4S-CS GAG.png]

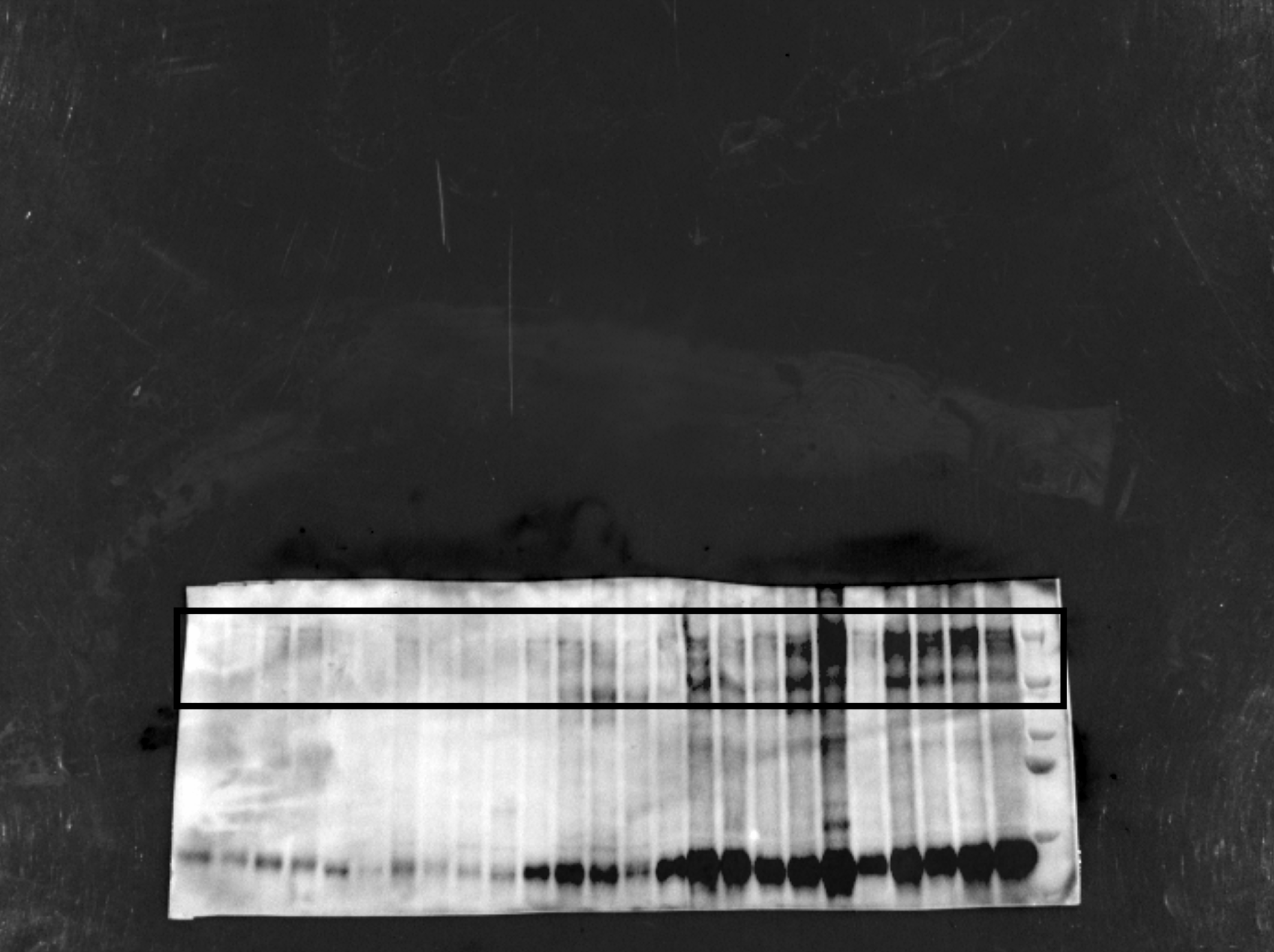

Supplement: Source data 1. [file elife-78387-data1.zip › Western blot source data/WB band outlined/Figure 5 - 4S-CS GAGs.tif]

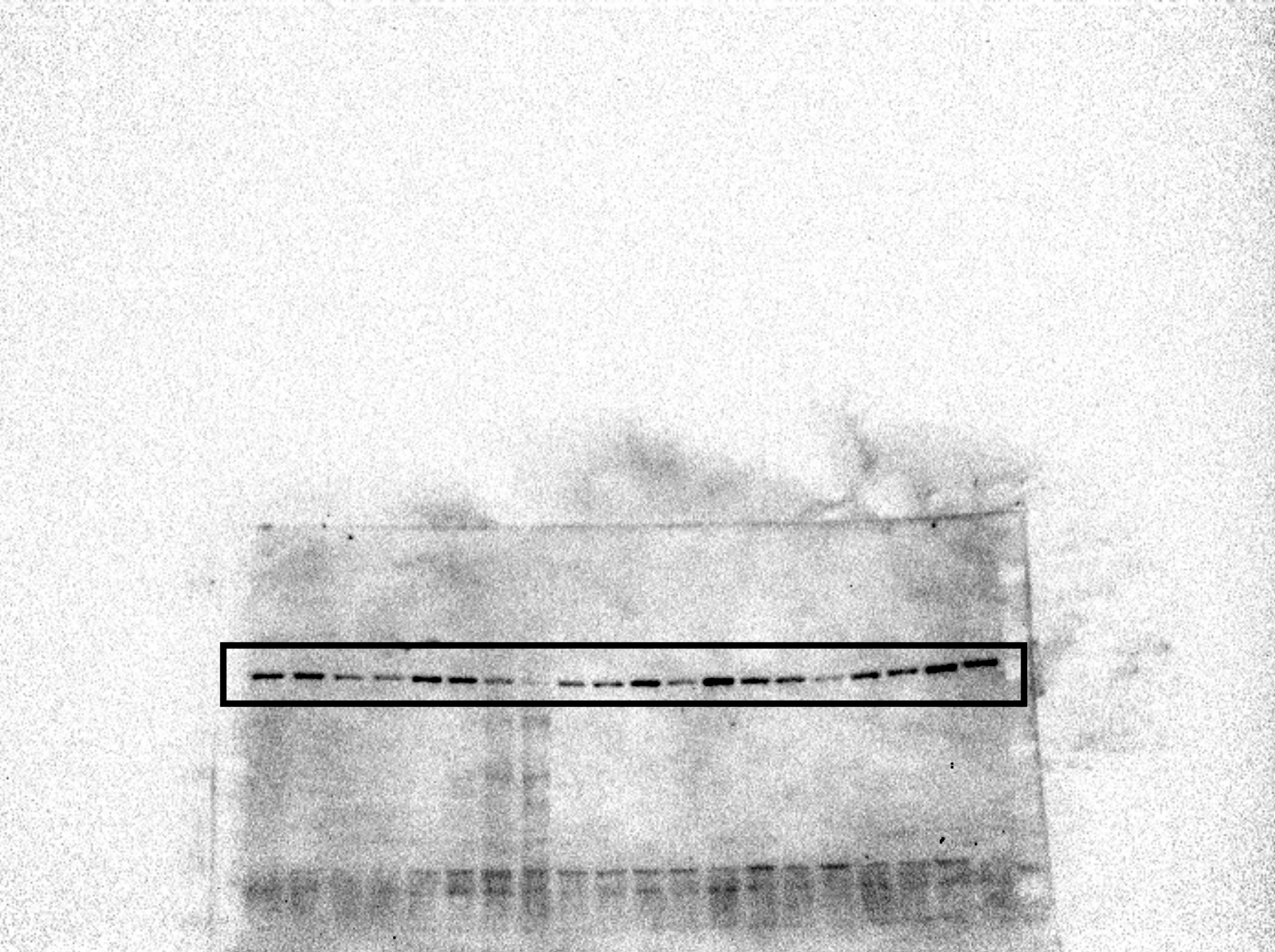

Supplement: Source data 1. [file elife-78387-data1.zip › Western blot source data/WB band outlined/Figure 4 - CHST11.tif]

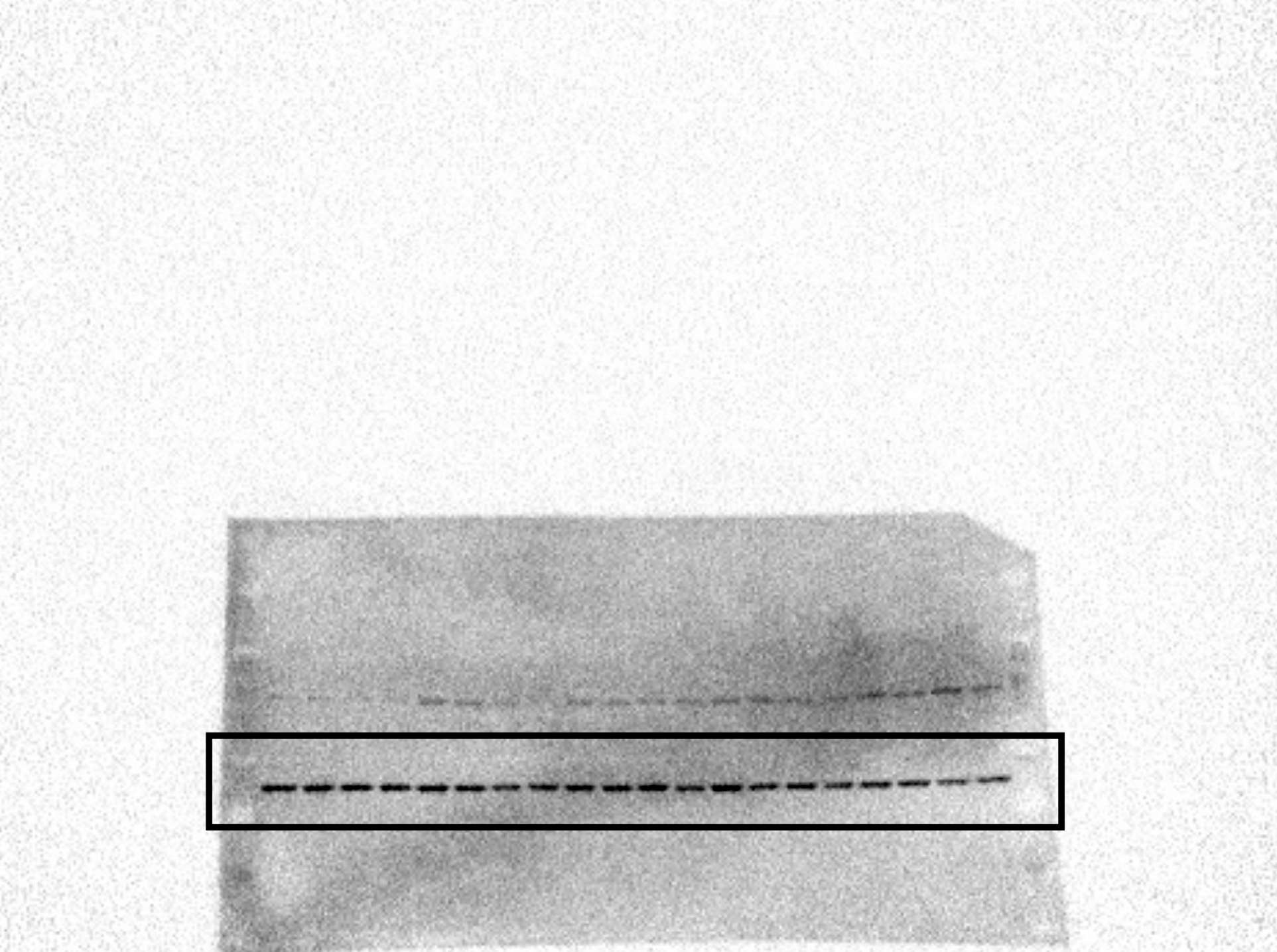

Supplement: Source data 1. [file elife-78387-data1.zip › Western blot source data/WB band outlined/Figure 4 - CHST15.tif]

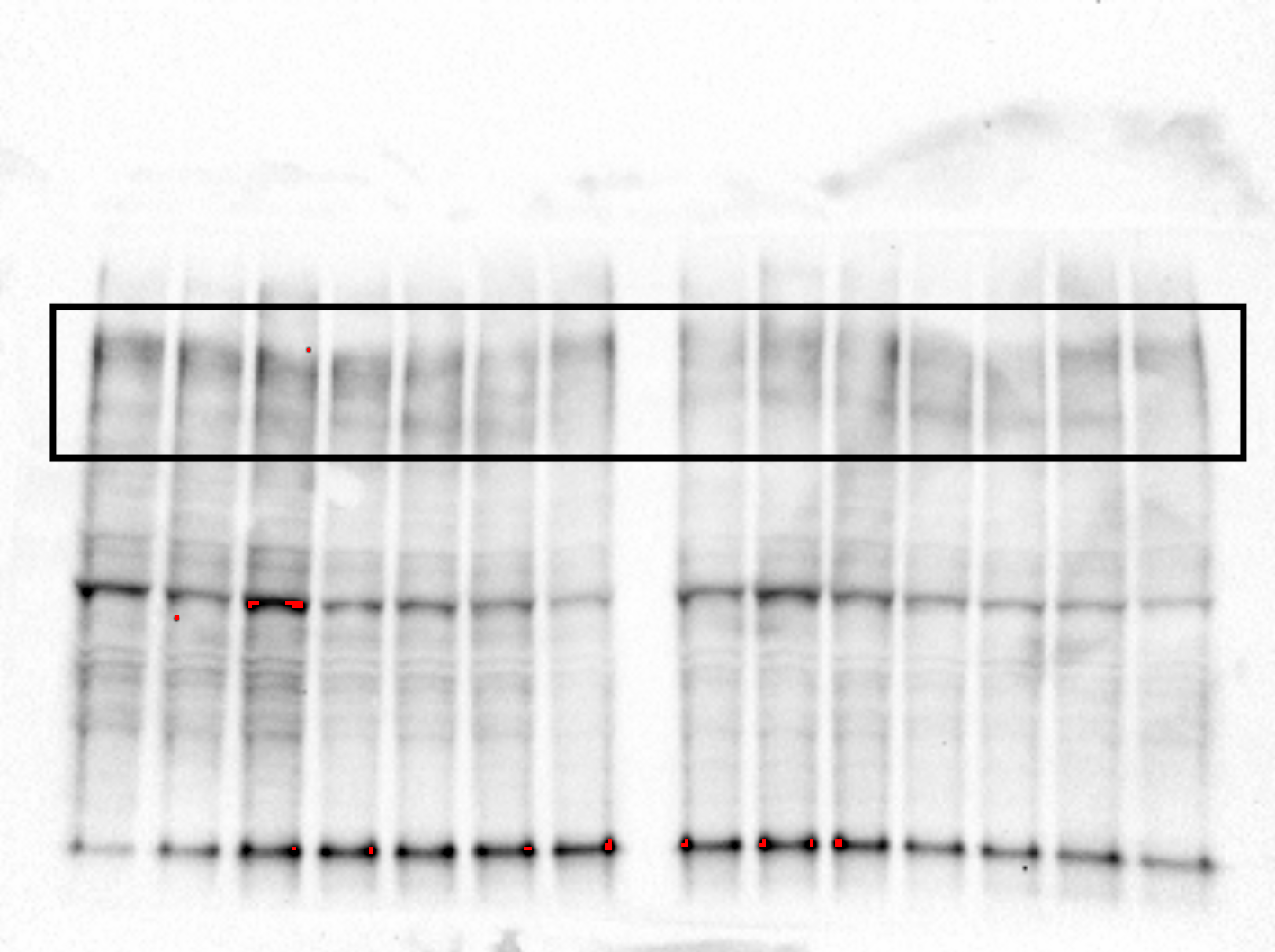

Supplement: Source data 1. [file elife-78387-data1.zip › Western blot source data/WB band outlined/Figure 6 - 6S-CS GAGs.tif]

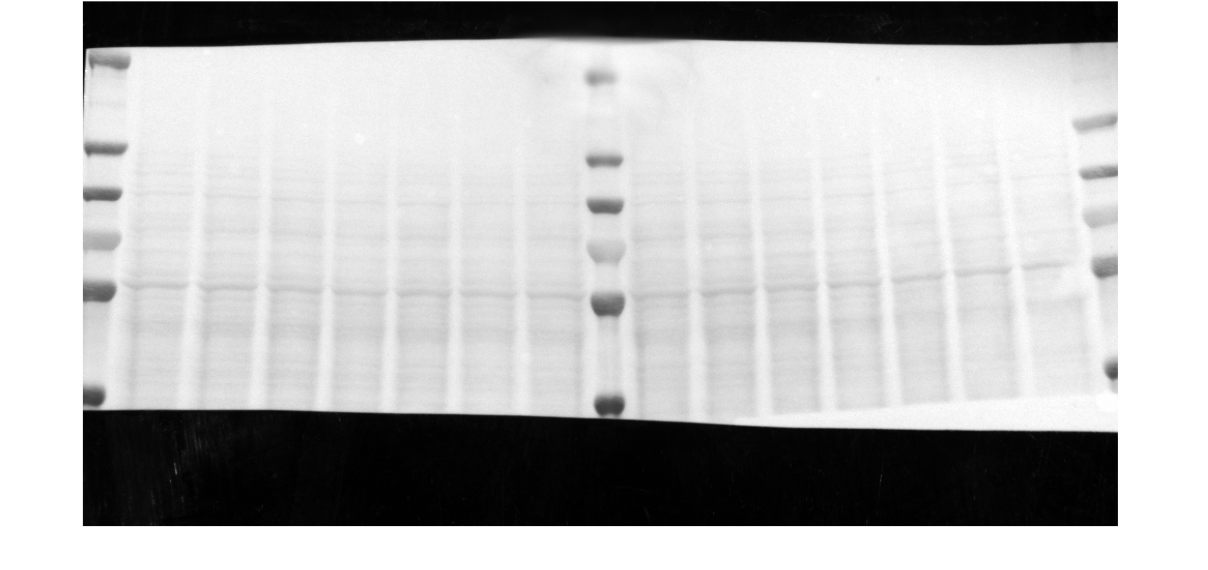

Supplement: Source data 1. [file elife-78387-data1.zip › Western blot source data/WB band outlined/Figure S3- ponceau CHST15.png]
