# Supplementary material for: Chondroitin sulfate proteoglycan 4,6 sulfation regulates sympathetic nerve regeneration after myocardial infarction
Source: eLife. 2022 May 23;11:e78387. doi: 10.7554/eLife.78387 (PMC9197393; doi:10.7554/eLife.78387)
Supplement: Supplementary file 1. [file elife-78387-supp1.pdf]

Supplemental Data:

**Chondroitin Sulfate Proteoglycan 4,6 sulfation regulates sympathetic nerve regeneration after myocardial infarction**

Blake MR[1], Parrish DC[1], Staffenson MA[1], Sueda S[1,2], Woodward WR[1], Habecker BA[1]

[1]Department of Chemical Physiology and Biochemistry, Oregon Health and Science University, Portland, OR, United States of America, 97239 [2]Portland State University EXITO Scholars Program, Portland State University, Portland, OR, United States of America, 97201

**Address for Correspondence:**
Beth A. Habecker PhD
Department of Chemical Physiology and Biochemistry, L334
Oregon Health and Science University
3181 SW Sam Jackson Park Rd
Portland, OR, USA, 97239

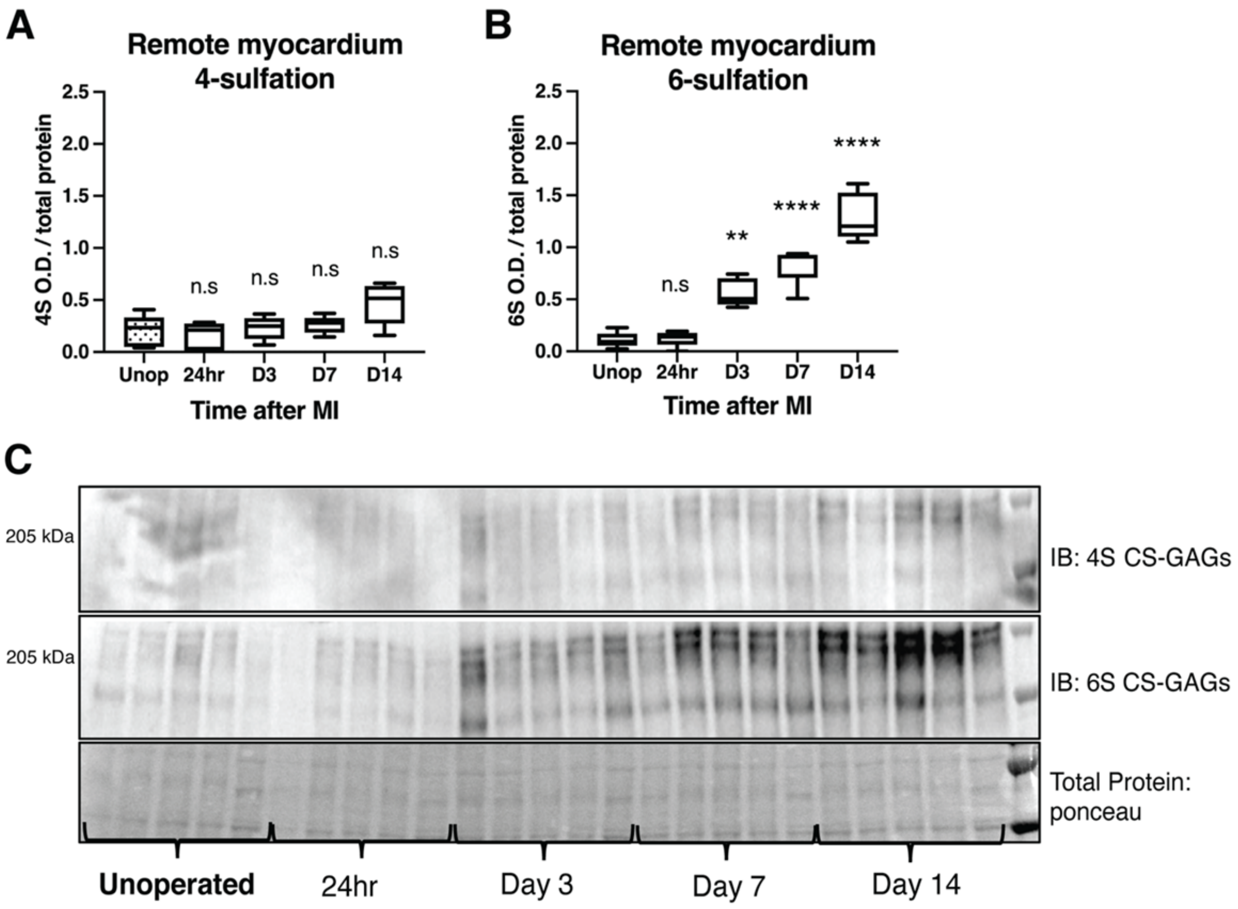


**Figure 1 - Figure Supplement 1:** Time course of CSPG sulfation in the remote myocardium (non-scar tissue) after MI caused by I/R. Western blot quantification of **(A)** 4-sulfation (4S CS-GAGs), Statistics: one-way ANOVA (Tukey's post-test), ns- not significant, p-value = 0.989, 0.997, 0.955, 0.053 respectively left to right, comparisons to unoperated tissue, n=5 animals per group. **(B)** Western blot quantification of 6-sulfation **(**6S CS-GAGs). Data are mean optical density (O.D.) ± SD. Statistics: one-way ANOVA (Tukey's post-test), ns- not significant p-value=0.999, **p-value=0.001, ****p-value<0.0001, comparisons to unoperated tissue, n=5 animals per group. **(C)** Example western blot images of A and B.

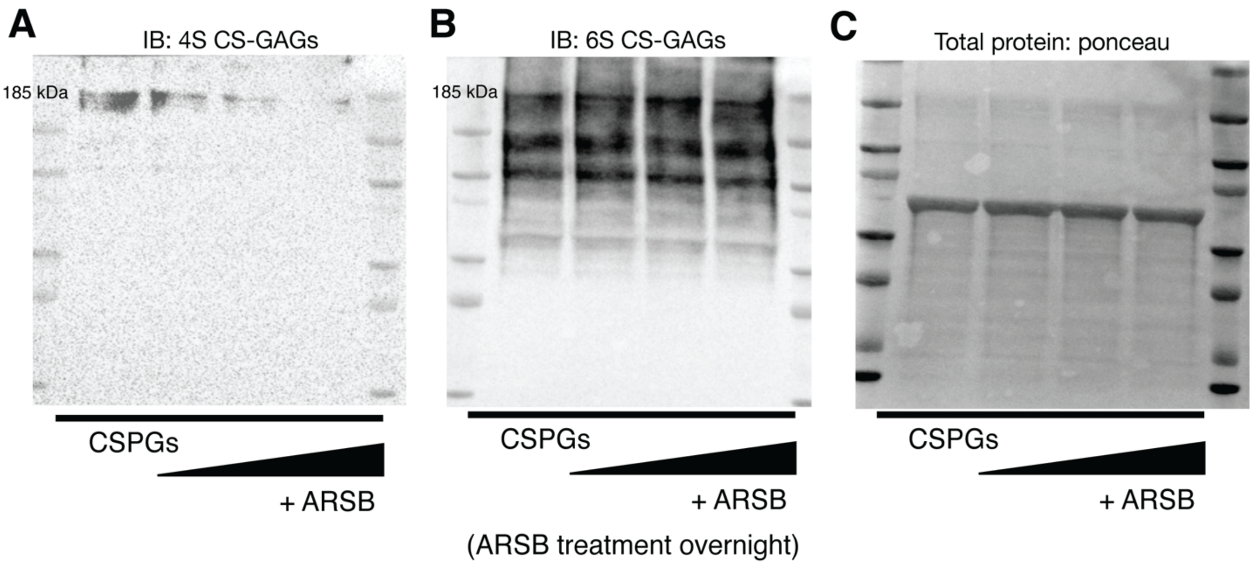


**Figure 2 - Figure Supplement 1:** ARSB removes 4S CS-GAGs and leaves 6S CS-GAGs intact. **(A)** Western blot of 4S content using purified CSPGs upon treatment of increasing concentrations of ARSB; vehicle, 0.3µg/mL, 0.6µg/mL, and 1.2µg/mL respectively left to right. **(B)** Western blot of 6S using purified CSPGs upon treatment of increasing concentrations of ARSB; vehicle, 0.3µg/mL, 0.6µg/mL, and 1.2µg/mL respectively left to right. **(C)** Total protein loaded of purified CSPGs treated with increasing concentrations of ARSB; vehicle, 0.3µg/mL, 0.6µg/mL, and 1.2µg/mL respectively left to right.

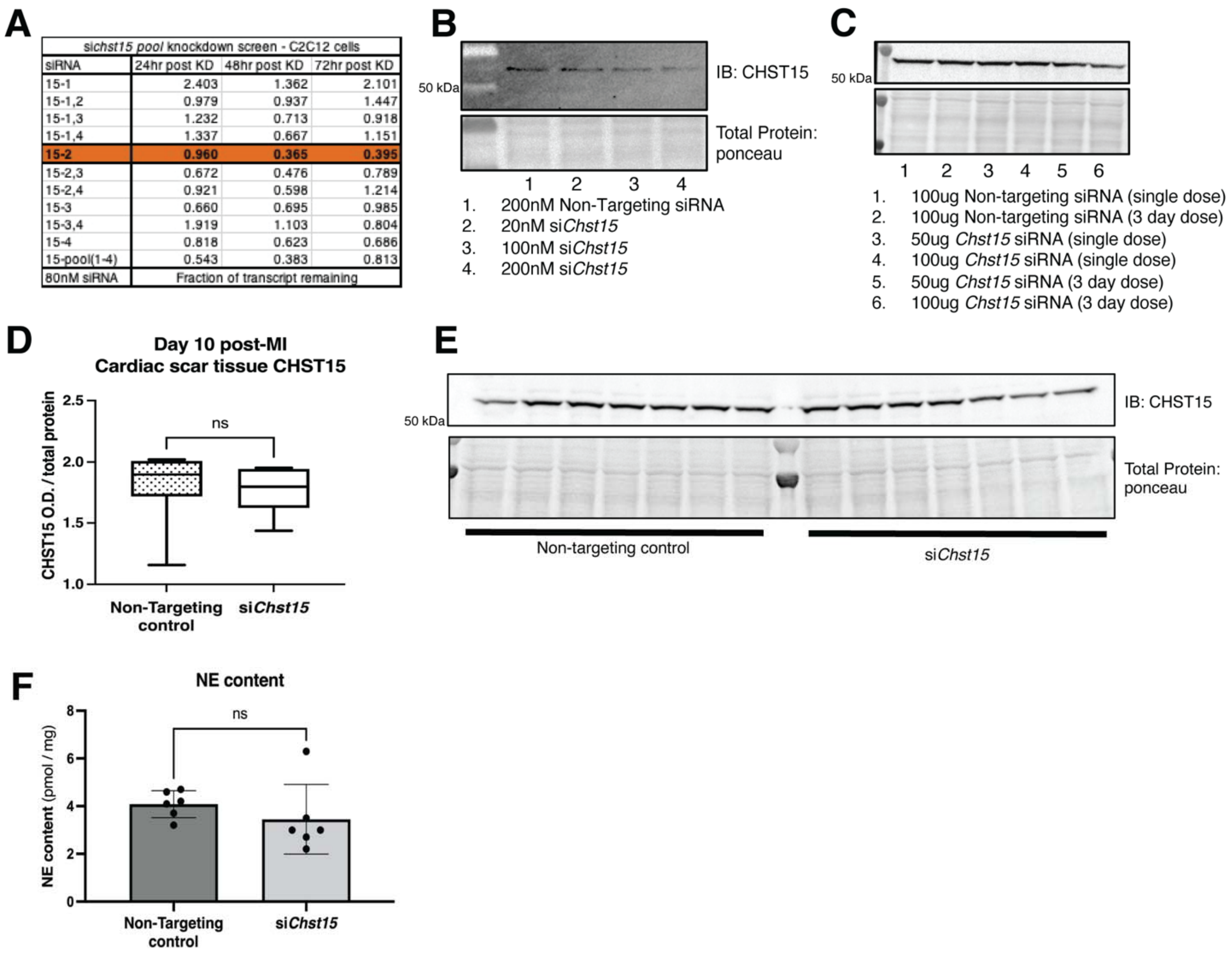


**Figure 6 - Figure Supplement 1:** Identification of an effective siRNA against *Chst15.* **(A)** qPCR knockdown of siRNA pool in C2C12 cells in 3 days after knockdown. Data shown fraction of transcript remaining, si*Chst15-2* was most effective in knockdown of *Chst15.* This transcript was selected for *in-vivo* studies **(B)** Western blot of CHST15 protein knockdown in C2C12 cells to confirm efficacy of si*Chst15-2,* comparison to Non-Targeting controls **(C)** Tail vein injection in mouse to determine ideal dosing for *in-vivo* si*Chst15* knockdown. Western blot of tail vein injection dosing trial for si*Chst15* with either 1 day or 3 days of injections. CHST15 protein in left ventricle (LV) 48hr after final tail vein injection, comparison to Non-targeting controls, all unoperated (non-MI) animals. **(D)** Full siRNA CHST15 experimental trial, CHST15 protein expression on D10 post MI quantified, siRNA injection D3, 5, 7 post-MI. Data are mean optical density (O.D.) ± SD, n=7 animals per treatment group, statistics; student t-test (Welch's test), ns – not significant, p-value=0.833. **(E)** Western blot of CHST15 protein expression D10 post-MI in siRNA treated animals. **(F)** NE content in the cardiac scar following siRNA treatment. NE was not increased with reinnervation, consistent with previous studies showing suppression of NE synthesis and reuptake by inflammatory cytokines (Parrish et al., 2010).

Quantification of n=6 animals for non-targeting controls and *Chst15 siRNA* treatment. Data are mean NE content ± SD. Statistics; student t-test (Welch's test), n.s.- not significant, p-value=0.345.
